# (AB)_*n*_ Star Block Polymers Derived from CO_2_: Influence of Architecture and Postpolymerization Modification

**DOI:** 10.1021/acs.macromol.5c01236

**Published:** 2025-10-13

**Authors:** Gregory S. Sulley, Kam C. Poon, Georgina L. Gregory, Charlotte K. Williams

**Affiliations:** Chemistry Research Laboratory, Department of Chemistry, 6396 University of Oxford, Oxford OX1 3TA, U.K.

## Abstract

The utilization of CO_2_ as a sustainable feedstock for oxygenated polymers offers a promising route to high-performance materials while addressing environmental challenges. This study investigates the synthesis of high-molar-mass, nonlinear polymer architectures using switchable catalysis, focusing on multiarm star block polymers derived from vinyl-cyclohexene oxide (vCHO), CO_2_, and ε-decalactone (ε-DL). A [Zn­(II)­Mg­(II)] organometallic catalyst and multifunctional chain-transfer agents (CTAs) are employed in a “core-first” approach to produce tri-, tetra-, and hexafunctional star block polymers. Thermomechanical and morphological properties were evaluated as a function of molar mass, number of arms, and architecture, indicating the differences between star and linear structures. Postpolymerization modification of the polycarbonate block, via thiol–ene chemistry, introduced pendant hydroxyl groups, enhancing hydrogen bonding and microphase separation, significantly impacting thermal and mechanical performance. This work highlights the versatility of switchable catalysis in accessing star polymers while underscoring the potential of integrating architectural control and functionalization to enhance the performance and applicability of CO_2_-derived poly­(ester-*b*-carbonate)­s.

## Graphical abstract

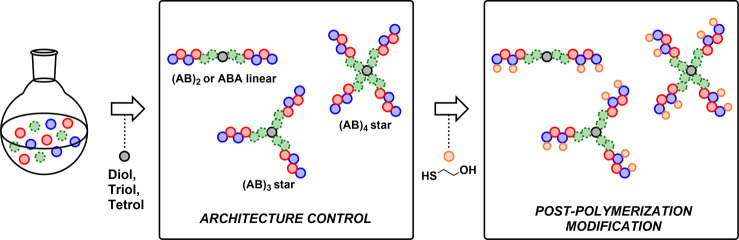

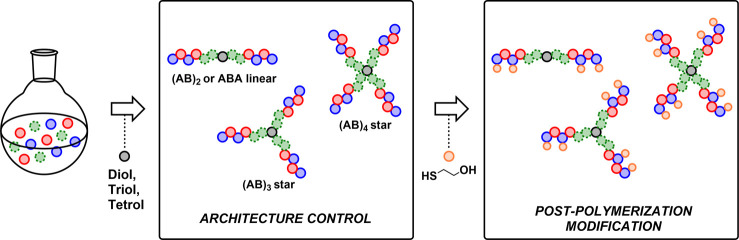

## Introduction

The increasing global demand for plastic is resulting in accelerating greenhouse gas (GHG) emissions from plastic production, use, and end-of-life recycling or disposal.^1^ Developing new sustainable alternatives to petrochemical incumbent materials will be critical to reducing the 1.8 Gt of carbon dioxide (CO_2_)-equivalents emitted annually from polymer manufacturing.^2–4^ Directly enchaining CO_2_ through ring-opening copolymerization (ROCOP) with epoxides to produce polycarbonates is an attractive means to reduce GHG emissions while utilizing this cheap, abundant, waste feedstock.^5–7^ The ROCOP of CO_2_ with epoxides allows for a 3-fold reduction in emissions relative to the corresponding polyether; for every molecule of CO_2_ polymerized, another two are saved by replacing the epoxide.^8–10^ Moreover, recent reports have demonstrated the effective chemical (closed-loop) recycling of these aliphatic polycarbonates to epoxides with high selectivity and rates, may help enable a circular plastic economy.^11–14^

Recently, it has been shown that coupling high-*T*_g_ CO_2_-derived polycarbonates with small amounts of low glass transition temperature (*T*_g_) polymers is an effective way to overcome the brittleness of the polycarbonates and produce toughened materials.^15,16^ So far, this was achieved through the formation of linear (i.e., a “2-arm star”) triblock polymers ((AB)_2_ or ABA type), where the outer A blocks are the hard, high-*T*_g_ polycarbonates flanking a soft, low-*T*_g_ block.^17^ By carefully tuning the overall molar mass and block ratios, the resulting material properties can be tuned to produce pressure-sensitive adhesives, elastomers, and plastics.^15,18,19^ Furthermore, postpolymerization functionalization of alkene-containing polycarbonates facilitated further tuning of thermomechanical properties.^20,21^ However, to date, the influence of block polymer architecture over the material properties of CO_2_-derived plastics has not been thoroughly explored or understood.^22^

Switchable catalysis enables the direct production of block polymers using one catalyst and a monomer mixture.^15,17^ It is generally employed with mono- or bifunctional alcohols (chain-transfer agents) to prepare block polymers with linear (e.g., AB or ABA-type) architectures.^17^ Yet, the controlled polymerization cycles that are accessed during lactone ring-opening polymerization (ROP) and epoxide/CO_2_ ring-opening copolymerization (ROCOP) can be initiated from multifunctional alcohol chain-transfer agents, which should allow access to nonlinear polymers, for example those with star architectures,^23–25^ and have the potential to expand the capability of the switch catalysis technique.^26^

There are reports of the synthesis and structure–property relationships of star polymers prepared via either cyclic ester ROP or epoxide/CO_2_ ROCOP.^23,25,27^ For instance, multiarm star poly­(ε-dl)-poly­(l-lactide) copolymers, at fixed molar mass and block ratio, showed improved mechanical properties as elastomers with an increasing number of arms.^25^ Hillmyer and coworkers reported the synthesis of a series of (AB)_*n*_ star block polymers comprised of poly­(l-lactide) A blocks and poly­(γ-methyl-ε-caprolactone) B blocks.^28^ Compared with the linear analogues, the star polymers exhibited enhanced tensile strength, tensile toughness, increased strain hardening, and reduced stress relaxation. These impressive mechanical properties were attributed to the multiple anchoring sites provided by the star polymers within the domains of the microphase separated structures. Hillmyer and coworkers later showed that ABC stars containing poly­(l-lactide) (B) and poly­(d-lactide) (C) blocks could undergo stereocomplexation within the crystalline B and C domains, further reducing chain pull-out under applied stress.^29^

Bates and coworkers reported upon miktoarm star polymers composed of poly­(l-lactide) and poly­(γ-methyl-ε-caprolactone)-*b*-poly­(l-lactide) arms, prepared using a grafting-through synthetic platform.^30^ The resulting stars had an average of three, six, or nine block arms attached to a single PLLA unit. As the number of block arms increased, the tensile toughness improved, while the Young’s modulus decreased consistent with a drop in PLLA volume fraction.

Most studies on switchable epoxide, CO_2_, and lactone polymerizations focus on catalysis and only provide proof-of-concept block polymer samples.^17^ For example, a Cr­(III)-salen “switch catalyst” was explored for propylene oxide ROP and propylene oxide/anhydride ROCOP with various multifunctional initiators, but the catalysis was not optimized for producing high molar mass or high-performance materials.^31^ There has been little investigation into the use of multifunctional chain transfer agents (CTAs) in switchable polymerization catalysis, particularly for synthesizing high molar mass, oxygenated star block polymers to study structure–property relationships.^31^ A leading example from Feng and coworkers focused on the synthesis of poly­(propylene ether carbonate)-*b*-poly­(cyclohexene phthalate) 4-arm star polymers.^32^ The resulting materials were found to exhibit heat resistance comparable to polyolefins and superior mechanical properties (ultimate tensile strength and elongation at break) compared to their linear block polymers with the same overall compositions.

This study investigates switchable polymerization catalysis using mixtures of ε-decalactone/vinyl cyclohexene oxide/CO_2_ with a [Zn­(II)­Mg­(II)] organometallic catalyst. ε-Decalactone provides low-*T*_g_ polyester blocks, which provides elasticity and ductility, while the high-*T*_g_ poly­(vinyl cyclohexene carbonate) blocks provide strength and rigidity.^15^ The noninitiating [Zn­(II)­Mg­(II)] catalyst was selected for its high rates of both ROP and ROCOP, selectivity, control, and end-group fidelity.^15^ A series of initiators featuring multiple hydroxyl groups was deliberately used to selectively target star block polymers. Additionally, using vinyl cyclohexene oxide should facilitate postpolymerization functionalization as a strategy to fine-tune block microphase separation and properties.

## Results

The use of polyhydroxy (*n*_OH_) species as both the initiator and chain-transfer agent (CTA) should result in the formation of star polymers, where the number of arms depends on the concentration/number of hydroxyl groups, *n*_OH_. Initially, the ROP of ε-decalactone (ε-DL) was performed using the [Zn­(II)­Mg­(II)] organometallic catalyst and the target multifunctional CTAs: tris­(hydroxymethyl)­propane (TMP, *n*_OH_ = 3) and pentaerythritol (PER, *n*_OH_ = 4) (Scheme S1). Using the conditions of catalyst:CTA:ε-DL of 1:4:200 at 80 °C ([ε-DL] = 1.7 M) resulted in polymerizations with TMP (*n*_OH_ = 3) and PER (*n*_OH_ = 4) that each achieved 44% and 95% ε-DL conversion after 15 min (TOF = 352 h^–1^) and 30 min (TOF = 380 h^–1^), respectively (Table S1). These polymerization studies demonstrate the independence of the rate with the initiator and served as a benchmark from which to further evaluate the tolerance of the catalyst to CTA and to determine the degree of functionalization on the various cores (Scheme 1).

**Scheme 1:**
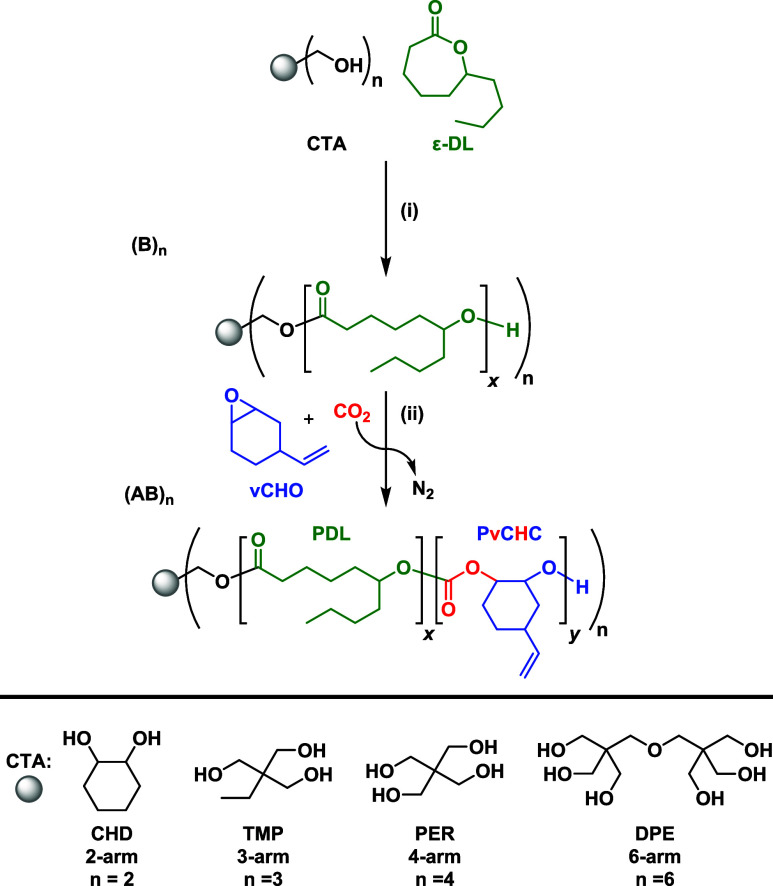
Synthesis of (AB)_*n*_ Star Block Polymers Comprising PDL-*b*-PvCHC Diblock Arms^a^

a (i) Synthesis of poly­(ε-decalactone): cat. = [Zn­(II)­Mg­(II)], cat.:CTA:ε-DL = 1:4:940, 80 °C, 1 h. (ii) cat.:vCHO = 1:950, CO_2_ (20 bar), 80 °C, 20 h.

The ^1^H NMR spectra for the purified multiarm star poly­(ε-decalactone) (PDL) polymers revealed characteristic signals corresponding to methine protons in the main polymer backbone (4.85 ppm) and end-group (3.58 ppm), and methylene protons on the CTA/initiating species (3.95–4.20 ppm) (Figure S1). Observation of these signals, after precipitation of the polymer into methanol, reassured us that initiation from the multifunctional initiator had occurred, given the good solubility of both CTAs in methanol. The relative integrals suggested complete arm functionalization, i.e., the formation of either 3- or 4-arm star polymers (Figures S2 and S3). The total degree of polymerization (DP_total_) for each star polymer was calculated from the relative integrals of the PDL backbone and end-group signals in a purified sample. DP_total_ was determined to be 27 and 62 for the three- and four-arm stars, respectively, in very good agreement with the values expected based on the conversion and initial monomer loading (Table S1).

The reaction of the polymer hydroxyl end-groups with [2-chloro-4,4,5,5-tetramethyl-1,3,2-dioxaphospholane] was used as a qualitative measure of end-group fidelity and core functionalization by observing the signals present in the ^31^P­{^1^H} NMR spectra (Figure S4). A diagnostic signal for the 2° hydroxyl chain-end in PDL was observed at 147.1 ppm, with no clear evidence of 1° hydroxyl groups arising from the starting alcohols. In some cases, minor baseline signals were observed downfield of the 2° hydroxyl; however, these were not due to unreacted hydroxyl groups on either TMP or PER cores and remain unassigned.

Analysis of the PDL star polymers by SEC revealed monomodal molar mass distributions with moderately low dispersity (*Đ*_M_ < 1.20) (Figure S5). A comparison between the molar mass by SEC and *M*_n_ calculated from the ^1^H NMR spectrum of the pure samples showed the value from SEC to be slightly higher in each case, which could be attributed to SEC calibration effects resulting from the use of poly­(styrene) standards.

Building from the promising data showing desired star polymer formation, switch catalysis was applied to prepare star block polymers, with each arm composed of poly­(ε-decalactone)-*b*-poly­(vinyl cyclohexene carbonate) ((PDL-*b*-PvCHC)_2_) (Scheme 1). To achieve this, the catalyst:CTA:ε-DL:vCHO loading was fixed at 1:4:940:950 (pCO_2_ = 20 bar), with the CTA selected depending on the desired number of arms. The ROP of ε-DL was followed by the ROCOP of vinyl cyclohexene oxide (vCHO) and CO_2_, fashioning a series of multiarm star block polymers with a soft (−60 < *T*_g_ < −30 °C) polyester interior of PDL blocks, a hard (70 < *T*_g_ < 90 °C) polycarbonate exterior of PvCHC blocks, and with a target block ratio of 50 wt % (*x*:*y* = 50:50, Table 1). At this stage, the synthesis of an additional 6-arm star block polymer was targeted by employing dipentaerythritol (DPE) as the CTA.

**Table 1:** (PvCHC-*b*-PDL)_*n*_ [(AB)_*n*_] Star Block Polymer Characterization Data

Entry	Polymer^a^	*n*_arms_ ^b^	*x*:*y* /wt %^c^	*M*_n,SEC_ ^d^ (kg mol^–1^) [*Đ*_M_]	*T*_g,DSC_ ^e^ (°C)	*T*_g,DMA_ ^f^ (°C)
1	(AB)_2_-63	2	49:51	66.3 [1.22]	–52	+91
2	(AB)_3_-52	3	50:50	36.0 [1.40]	–38	*n.d.*
3	(AB)_3_-88	3	52:48	75.1 [1.18]	–55	+71
4	(AB)_4_-90	4	52:48	79.9 [1.34]	–63	+73
5	(AB)_6_-72	6	51:49	63.4 [1.34]	–32	*n.d.*

a (AB)_*n*_-#; where A = PvCHC, B = PDL, *n* = number of arms, # = peak molar mass of the major distribution (SEC).

b Number of arms based on full functionalization from CTA.

c Relative block weight fraction, determined from ^1^H NMR spectroscopy (Figures S6–S11).

d Molar mass determined from SEC, *Đ*_M_ = *M*_w_/*M*_n_.

e Glass transition temperature determined from DSC (−90 to 120 °C), taken as the midpoint of transition on the third heating cycle (Figures S23–S27).

f Upper glass transition temperature determined from DMA temperature sweep (30–110 °C), taken as the peak maxima in tan­(δ) (Figures S32–S35). n.d. = glass transition temperature not determinable (samples deforming to the limits of the geometry before the *T*_g_).

The block ratio of 50 wt % was preferred given the promising thermomechanical properties observed in linear ABA triblock polymers of PCHC-*b*-PDL-*b*-PCHC at 50 wt % and also allowed for high utilization of CO_2_ in the resulting materials.^15^ The pendent vinyl moieties provided by the vCHO monomer are located in the polycarbonate outer blocks, offering the opportunity to later modify them, e.g., by thiol–ene reactions. The star block polymers are denoted (AB)_*n*_-#, where A = polycarbonate outer block, B = polyester inner block, *n* = number of arms, and # = molar mass by SEC.

To determine the monomer selectivity, a model polymerization was run using 1 bar of CO_2_ pressure and monitored using *in situ* ATR-IR spectroscopy and SEC aliquots. The data showed the expected catalyst selectivity, initially for ε-DL ROP, followed by a switch to vCHO/CO_2_ ROCOP after the introduction of a CO_2_ atmosphere, and was substantiated by an increase in the molar mass of aliquot samples taken at key stages during the reaction and by the retention of low dispersity (Figure 1). Larger-scale polymerizations to prepare high molar mass star block polymers with varying arm numbers were performed under 20 bar of CO_2_ pressure using a stainless steel Parr reactor. Under these conditions, the polymerizations reach high vCHO monomer conversion (>95%, TON > 900, TOF > 38 h^–1^) within a reasonable time frame (<24 h).

**Figure 1:**
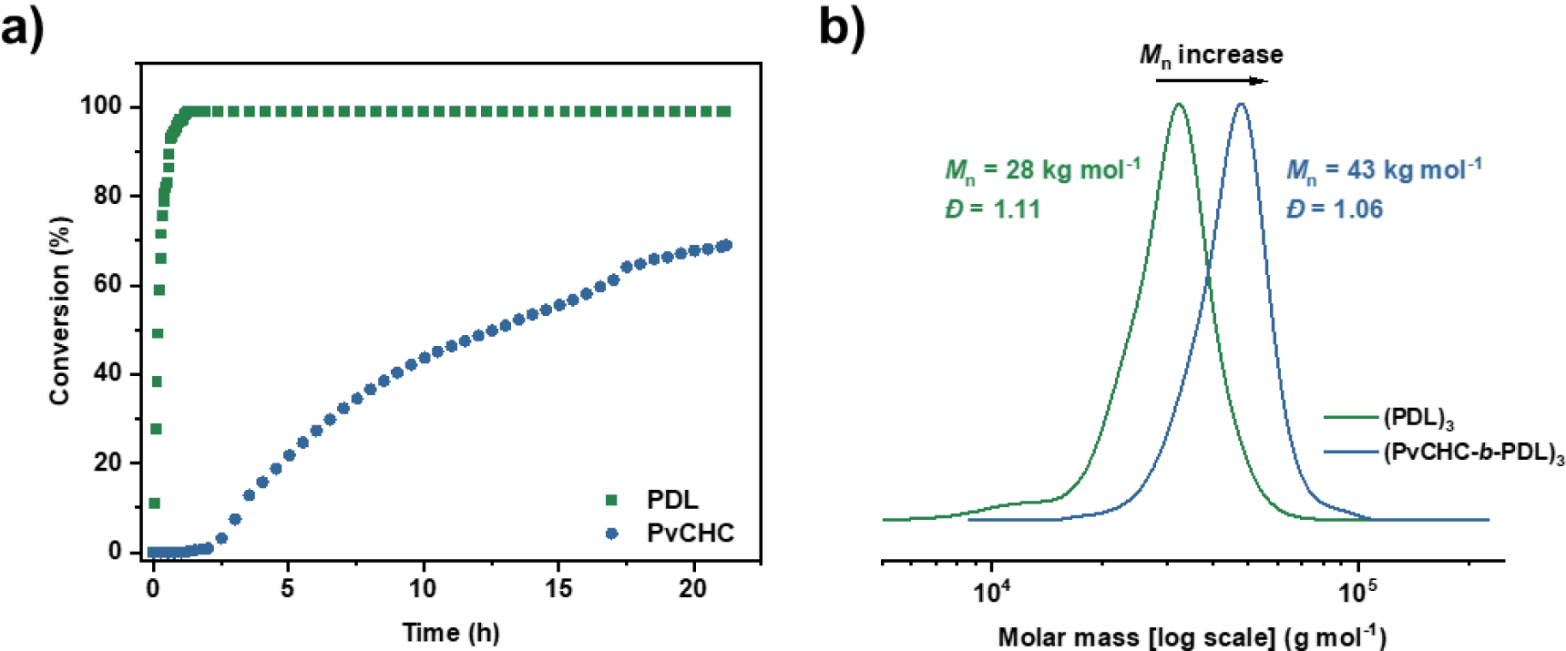
a) *In situ* ATR-IR spectroscopy reaction monitoring for the synthesis of (PvCHC-*b*-PDL)_3_ star block polymer. b) SEC aliquots taken under N_2_ (green) and CO_2_ (blue) atmosphere reaction stages.

The high monomer conversions (>95%) reached in each polymerization resulted in the final block compositions being representative of the starting monomer stoichiometry. As such, the target block ratio of ∼50 wt % was achieved in all samples, as determined by comparing the relative integrals for the PDL side chain methyl signal (0.88 ppm) and PvCHC side chain vinyl C*H* signal (5.88–5.63 ppm); signal overlap prevented the typical use of backbone polymer signals corresponding to PDL (4.85 ppm) and PvCHC (4.77 ppm) methine signals (Figures S6–S11).

Analysis of the star block polymers by SEC, equipped with an RI detector, revealed trimodal molecular molar mass distributions (Figures S12 and S13, Table S2). This observation was surprising, given that the PDL homopolymers clearly show monomodal molar mass distributions. In each case for the block stars, the major molecular weight distribution matches closely to the target molar mass, with a smaller secondary distribution at lower molar mass and a shoulder at higher molar mass. These minor distributions might arise from residual protic impurities present in the monomers (lower molar mass) or perhaps from aggregation in solution, resulting from the covalent cross-linking at the core of the star polymer, as well as star–star coupling occurring during transesterification at high conversions (higher molar mass).^23^

To confirm the purity and architecture of the star block copolymers, we employed diffusion-ordered spectroscopy (DOSY) NMR and visco-equipped size exclusion chromatography (SEC). DOSY NMR revealed a single, well-defined diffusion coefficient for each sample, indicating compositional homogeneity and the absence of significant low- or high-molecular-weight impurities (Figures S14–S18). SEC traces of the 2- and 3-arm materials were monomodal, while the 4- and 6-arm stars showed minor shoulders, attributed to trace quantities of protic impurities during ROP and ROCOP. The observed protic impurities are minimal and likely arise from residual moisture introduced during the recrystallization of PER and DPE initiators from water. Intrinsic viscosity (IV_*n*_) data provided further architectural insight: all star polymers exhibited viscosities lower than those of the linear analogue of similar molar mass ((AB)_2_-63), and IV_*n*_ decreased systematically with increasing arm number, consistent with increasingly compact, branched conformations (Figure S19). These results confirm that the dominant species in all samples is a well-defined star polymer.

End-group analysis of the star block polymers, by ^31^P­{^1^H} NMR spectroscopy, showed only the multiplet corresponding to PvCHC secondary hydroxyl chain ends (146.9–146.4 ppm) (Figure S20).^33^ Signals corresponding to the primary CTA alcohols and the sharp singlet corresponding to PDL hydroxyl chain ends (147.1 ppm) were absent, which indicated complete initiator consumption and efficient block polymer formation (Figure S21).

All star block polymers were thermally stable, with the temperature at 5% mass loss (*T*_d,5%_) exceeding 250 °C in all cases (Table S3 and Figure S22). Thermal characterization of the star block polymers identified two glass transition temperatures at values consistent with those of the constituent blocks (Figures S23–S27). DSC showed that the low temperature *T*_g_ corresponds to the PDL blocks at values from −52 to −63 °C for linear, 3-arm (88 kg mol^–1^), and 4-arm samples, respectively (Table 1, entries 1, 3–4). The lower molar mass 3-arm (52 kg mol^–1^) and 6-arm samples exhibited broad transitions at −38 °C and −32 °C, respectively, indicating partial block miscibility (Table 1, entries 2, 5). The *T*_g_ for PvCHC (expected around 100 °C) was difficult to observe by DSC but could instead be identified for all but two samples using DMA by taking the peak maxima in tan­(δ). The 3-arm (52 kg mol^–1^) and 6-arm samples were not analyzed by DMA due to difficulty in processing samples suitable for the measurement, likely a result of their lower overall molar mass. The linear (2-arm star block) sample showed the highest upper *T*_g_ at 91 °C, close to the value expected for pure PvCHC (118 °C). Star block polymers (AB)_3_-88 and (AB)_4_-90 showed upper *T*_g_ values of around 70 °C, somewhat lower than those of either the block or PvCHC polymer, which may indicate some degree of block miscibility. In order to ensure complete block phase separation, polymers with higher molar masses than can currently be readily accessed in this field would be required. Therefore, a reasonable compromise was required between polymer synthetic accessibility and producing phase-separated samples, particularly those exhibiting long-range order.

The effect of molar mass and arm number on the mechanical properties of the star block polymers was investigated with tensile mechanical testing. Specimens were prepared, where possible, from solvent-cast films (30 wt % THF) and subjected to uniaxial extension experiments according to ISO 527 type 5b (10 mm min^–1^). It is important to note that homopolymers of both PDL and PvCHC are challenging to process at equivalent molar masses.^15,34^ For each material, five repeat experiments were conducted, and the average values and errors for the Young’s modulus, tensile strength, strain at break, and tensile toughness were determined (Table 2).

**Table 2:** (AB)_*n*_ Star Block Polymer Mechanical Characterization Data

Entry	Polymer	*M*_n,total_ ^a^	*M*_n,arm_ ^b^ (kg mol^–1^)	*E*_y_ ^c^ (MPa)	*σ*_y_ ^d^ (MPa)	ε_y_ ^e^ (%)	**σ^f^** (MPa)	**ε_b_ ^g^** (%)
1	ABA-50^h^	59.8 [1.10]	29.9	238 ± 35	6.7 ± 0.3	9.0 ± 1.7	20 ± 2	900 ± 104
2	(AB)_2_-63	66.3 [1.22]	31.7	212 ± 12	9.9 ± 0.3	9.9 ± 0.3	19 ± 1.7	1167 ± 147
3	(AB)_3_-52	36.0 [1.40]	17.3	7.2 ± 0.1	*n.d.*	*n.d.*	0.7 ± 0.07	663 ± 186
4	(AB)_3_-88	75.1 [1.18]	29.3	78 ± 9	4.8 ± 0.5	14 ± 0.6	12 ± 0.5	1500 ± 219
5	(AB)_4_-90	79.9 [1.34]	22.5	25 ± 7	1.7 ± 0.3	33 ± 9	6.8 ± 0.4	1678 ± 195
6	(AB)_6_-72	63.4 [1.34]	12.0	*n.d.* (unable to process free-standing film)				

a Molar mass data for the entire chromatogram without peak deconvolution.

b Calculated as (*M*_n,SEC_)/*n*, where *n* is the number of arms or AB segments, assuming full core functionalization and equal arm lengths.

c Young’s modulus.

d Yield stress.

e Yield strain.

f Tensile strength.

g Elongation at break. Average values and standard deviation from a minimum of 5 specimens. n.d. = not determined.

h Linear ABA triblock sample comprised PCHC-*b*-PDL-*b*-PCHC at 50 wt % relative block ratio.^14^

The block polymer, (AB)_2_-63, serves as a benchmark from which to compare the higher-arm star block polymers. It also enables a direct comparison to the analogous linear ABA triblock polymer (ABA-50), previously reported, in order to identify any effects of changing the hard block segments from PCHC to PvCHC. Both ABA-50 and (AB)_2_-63 possess 50 wt % hard block and have similar molar masses of 59.8 and 63.3 kg mol^–1^, respectively. It is anticipated that the pendent vinyl moieties of PvCHC should soften these materials compared with those with PCHC.

Tensile mechanical analysis conducted on (AB)_2_-63 showed an overall stress–strain profile similar to that of ABA-50 (Figure 2a). The Young’s modulus was comparable between ABA-50 and (AB)_2_-63 (238 vs. 212 MPa), indicating similar material stiffness within the elastic regime. The relative hard/soft block ratio of 50 wt % for (AB)_2_-63 resulted in it displaying properties akin to ductile plastic with a well-defined yield point visible on the curve below 10% strain. Beyond this point, the material experienced plastic deformation like ABA-50.

**Figure 2:**
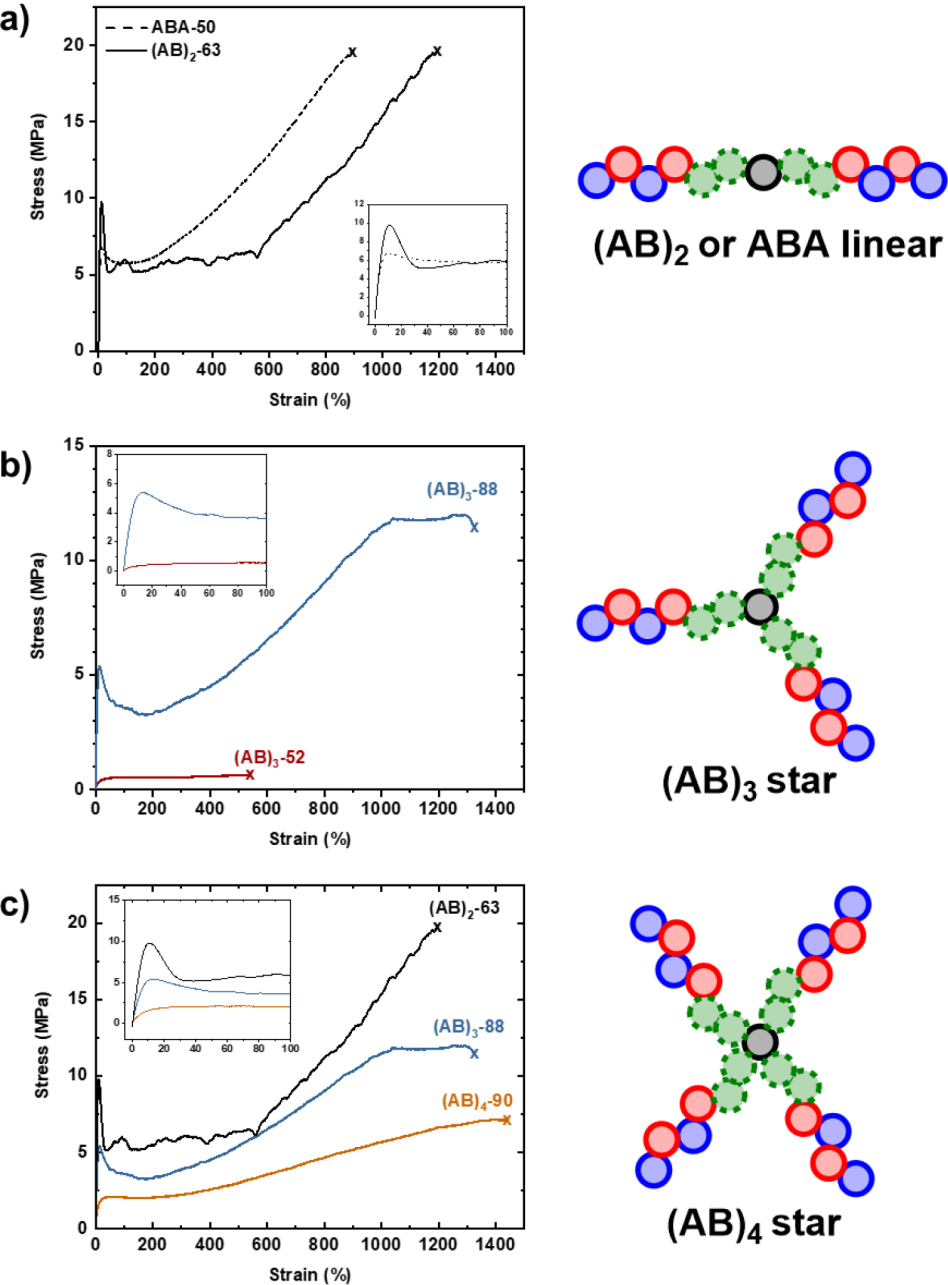
Representative stress–strain curves for a) linear triblock samples ABA-50 (50 wt % PCHC)^15^ and (AB)_2_-63 (50 wt % PvCHC^1^), b) 3-arm star block polymers of low (52 kg mol^–1^) and high (88 kg mol^–1^) molar mass, and C) high molar mass star block polymers with 2, 3, and 4 arms. Failure points are marked with an “X”. Insets: enlargement of the 0–100% region.

Most notably, there was a lengthened region of drawing or localized stretching (ε: 50–550%) in which polymer chains aligned parallel to the direction of extension, and the sample’s elongation increased substantially with minimal increase in stress. The chain alignment resulted in strain hardening, with sharply increasing stress until the point of fracture, as was observed for ABA-50 but with an earlier onset. The tensile strengths of the materials were roughly equivalent at around 20 MPa, but (AB)_2_-63 reached higher elongation before failure (1167% vs 900%). It is worth noting that the specimens possessed visual similarities during uniaxial extension; (AB)_2_-63 showed more whitening after entering the strain-hardening region, with almost the entire area under load becoming opaque in an identical fashion to ABA-50 (Figure S28). The 3-arm star (AB)_3_-52 was soft and highly flexible, almost at the threshold of easy manipulation outside of the mold. Uniaxial extension experiments showed characteristically soft plastic behavior. The specimens had a low Young’s modulus (*E*_y_ = 7.2 MPa), a diffuse yield point, and low resistance to plastic deformation, ending with failure at very low tensile stress (σ < 1 MPa) and moderate strain (ε_b_ = 663%) (Table 2, entry 3, and Figure 2b).

In contrast, when the molar mass of the 3-arm star polymer was increased to 88 kg mol^–1^, the mechanical properties were greatly improved. This may be expected, given that increased molar mass usually improves mechanical performance; however, it can sometimes be at the expense of processing capability. Fortunately, (AB)_3_-88 was still readily processed via solvent-casting methods and produced easy-to-handle, free-standing films. The increased molar mass resulted in a 10-fold increase in the Young’s modulus and a more defined yield point (σ_y_ = 4.8 MPa, ε_y_ = 14%) which in turn provided a more reliable boundary in which the material should exhibit elastic behavior. After yielding in the low strain region, (AB)_3_-88 displayed enhanced ductility with a large period of plastic deformation that included a region of strain hardening, ultimately reaching a tensile stress of 12 MPa before necking and failing at 1500% elongation. Based on the marked enhancement to both the thermal and mechanical properties, subsequent star block polymers aimed to achieve molar masses exceeding the original 50 kg mol^–1^ target.

The four-arm star sample, (AB)_4_-90, had around the same molar mass as (AB)_3_-88 but it showed a lower tensile strength of 6.8 MPa (Table 2, entry 5). The lower Young’s modulus also indicated a softer material, and the shape of the stress–strain profile suggested somewhat reduced ductility and toughness (Figure 2c). The sample still exhibited strain hardening, but to a lesser degree (above 300%).

Neither (AB)_3_-88 nor (AB)_4_-90 exhibited any stress-whitening, which set them apart from the linear block polymer analogue and suggested a direct benefit of the change in molecular architecture, given that specimen size, thickness, and processing conditions were identical (Figures S28 and S29). One clear advantage is the retention of optical clarity and transparency at stresses above the yield point. On the other hand, the presence of stress-whitening is often a good visual indicator of high stress or fatigue in materials, usually preceding failure, and can also provide some improvement in stress dissipation. In any case, the potential to alter the fracture dynamics of these polymers through a change in the architecture could be a useful feature.

Overall, (AB)_2_-63 was mechanically superior to all of the multiarm star polymers, despite having a lower overall molar mass compared to all but (AB)_3_-52. Indeed, the six-arm star sample, (AB)_6_-72, was unable to form a free-standing film, even though it has a high molar mass (72 kg mol^–1^).

The mechanical performance of the star block copolymers is strongly influenced by the ability of the midblock and end blocks to participate in physical or topological network formation. The entanglement molar mass (*M*_e_) of the rubbery PDL midblock is approximately 5.9 kg mol^–1^,^35^ and for the 6-arm star (AB)_6_-72, the PDL arms are ∼6 kg mol^–1^—at best marginally entangled. In contrast, the PDL block length increases to ∼12, ∼15, and ∼16 kg mol^–1^ in the 4-, 3-, and 2-arm stars, respectively, allowing for more effective midblock entanglement and improved mechanical integrity. Meanwhile, the glassy PvCHC outer blocks have a much higher *M*_e_ (45–62 kg mol^–1^),^34^ and the arm lengths in all cases fall well below this threshold, meaning that the PvCHC domains do not contribute via entanglement. Instead, they function as physical cross-links due to microphase separation into rigid domains. The poor mechanical performance and inability of (AB)_6_-72 to form robust free-standing films can therefore be attributed to insufficient entanglement in the PDL midblocks. These findings underscore the importance of balancing arm length and block identity to achieve mechanically stable star block copolymers.

The tensile mechanical data suggest that there may be a limiting molar mass for mechanical robustness specific to these particular polyester–polycarbonate block polymers. Considering the arm molar mass (*M*_n,arm_) allows for comparison between different arm numbers and, therefore, architectural variance (Figure 3). As expected, with increasing *M*_n,arm_, tensile stress and Young’s modulus increased. Elongation at break decreased somewhat with increasing *M*_n,arm_ for the higher molar mass samples, although the values remained in excess of 1000% and also had the largest statistical errors. The low ε_b_ observed for (AB)_2_-52 could be a result of ineffective stress distribution owing to incomplete microphase separation in the sample.

**Figure 3:**
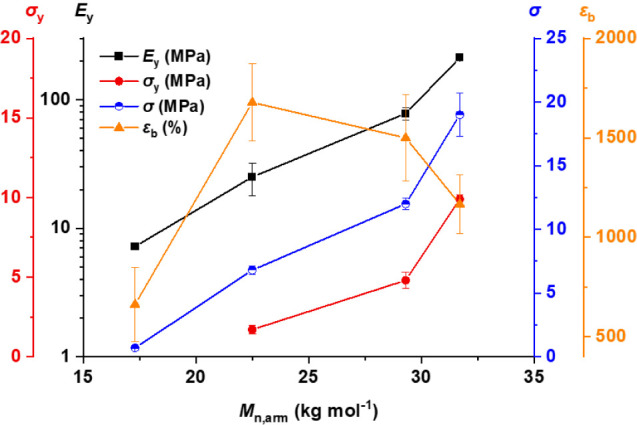
Trends in yield stress (σ_y_), Young’s modulus (*E*_y_), tensile stress (σ), and elongation at break (ε_b_) with respect to arm molar mass (*M*_n,arm_) across the range of (AB)_*n*_ star block polymers.

In the case of these (AB)_*n*_ star block polymers, for the mechanical performance of a sample with higher *n* to equal or surpass that of a lower *n* sample then it is likely that *M*_n,arm_ must be equivalent across samples or possibly even higher with increasing values of *n*. Therefore, as *n* increases, *M*_n,total_ must also increase to achieve the same mechanical performance. Such a concept was previously identified for (PSt-*b*-PIB)_5–21_ star polymers and has since been observed for other polymers, yet some reports suggest it is not a general rule.^25,36–38^ Nonetheless, the overall molar masses of these PvCHC-*b*-PDL star polymers are limited by virtue of the catalyst and/or conditions. Empirically, an overall molar mass of ∼63 kg mol^–1^ for a linear sample was sufficient to provide the best mechanical performance, although it should be noted that this is not the proven lower bound. Nonetheless, it equates to an *M*_n,arm_ of 31.5 kg mol^–1^, which gives an estimate for the minimum overall *M*_n_ for analogous 4- and 6-arm samples of 126 and 189 kg mol^–1^, respectively. Currently, both slow rates of polymerization and worsening catalyst tolerance at the high monomer loadings required to access molar masses as high as these have yet to be achieved for these block polymers, regardless of arm number.

Another strategy to enhance the performance of these star polymers is postpolymerization modification.^39^ Here, thiol–ene click chemistry was employed to install pendant hydroxyl moieties onto the polycarbonate block of each arm (Scheme 2). It was hypothesized that this should increase block immiscibility and may reinforce the hard domains through hydrogen bonding.^21,40^

**Scheme 2:**
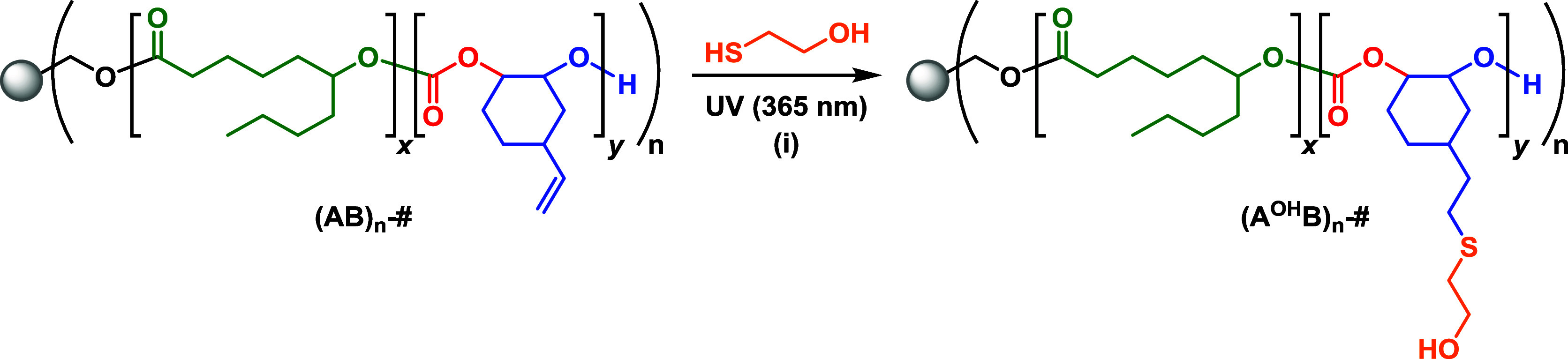
Postpolymerization Modification of (AB)*_n_* Star Block Polymers[Fn sch2-fn2]

a Reaction conditions (equiv. with respect to C=C moiety): (i) 2-mercaptoethanol (4 equiv.), 2,2-dimethoxyphenylacetophenone (DMPA) (0.4 equiv.), THF, UV (365 nm), 2 h.

The resulting modified star polymers were denoted (A^OH^B)_*n*_-#, where A^OH^ = PvCHC functionalized with 2-mercaptoethanol, B = PDL, *n* = number of arms, and # = molar mass, in this case, of the precursor star polymer for ease of comparison. It should also be noted that after functionalization, the relative hard block weight fraction increases to approximately 60 wt %.

Thermal characterization of the modified star polymers by DSC revealed two glass transition temperatures: one at low temperature (−47 to −49 °C) and one at high temperature (+59 to +76 °C), corresponding to the PDL and hydroxyl-functionalized PC blocks, respectively (Table S4). Compared to the starting block polymers, the more pronounced upper transitions suggest improved microphase separation, particularly in samples (A^OH^B)_3_-52 and (A^OH^B)_6_-72 where the precursors showed block miscibility. Thermal stability decreased slightly upon functionalization; however, the *T*_d,5%_ remained above 200 °C for all functionalized materials (Table S3 and Figure S30).

To investigate any potential for transesterification by the pendent hydroxyl group, (A^OH^B)_3_-88 was heated above the order–disorder temperature (120 °C), under 1 ton m^–2^ pressure, for 30 min (three times longer than required to reprocess the material). Addition of a phospholane reagent followed by ^31^P­{^1^H} NMR spectroscopy revealed that only PvCHC end-group and the mercaptoethanol environments were observed at 146.8 and 147.9 ppm, respectively (Figure S31). Critically, no signals were observed at 147.1 ppm, corresponding to the 2° hydroxyl chain-end of PDL, suggesting no transesterification had occurred over the course of the compression molding.

Dynamic mechanical analysis (DMA) temperature sweeps, from 30 to 110 °C, showed further evidence of the PC upper glass transition (Table S3). The peak maxima in tan­(δ) (i.e., upper *T*_g_ values) were consistently at higher values than the equivalent peaks for the precursor star polymers (Figures S32–S35). This is clear when comparing (AB)_3_-88 and (A^OH^B)_3_-88 with *T*_g_ values of 71 and 95 °C, respectively (Figure 4a). Additionally, postfunctionalization helped improve the thermo-mechanical stability, as identified by the extended plateau region of the storage modulus (*E*′) up to 80 °C prior to a decrease in *E*′. This indicated that the operating temperature window of the modified star block polymers is widened compared with the nonmodified variants.

**Figure 4:**
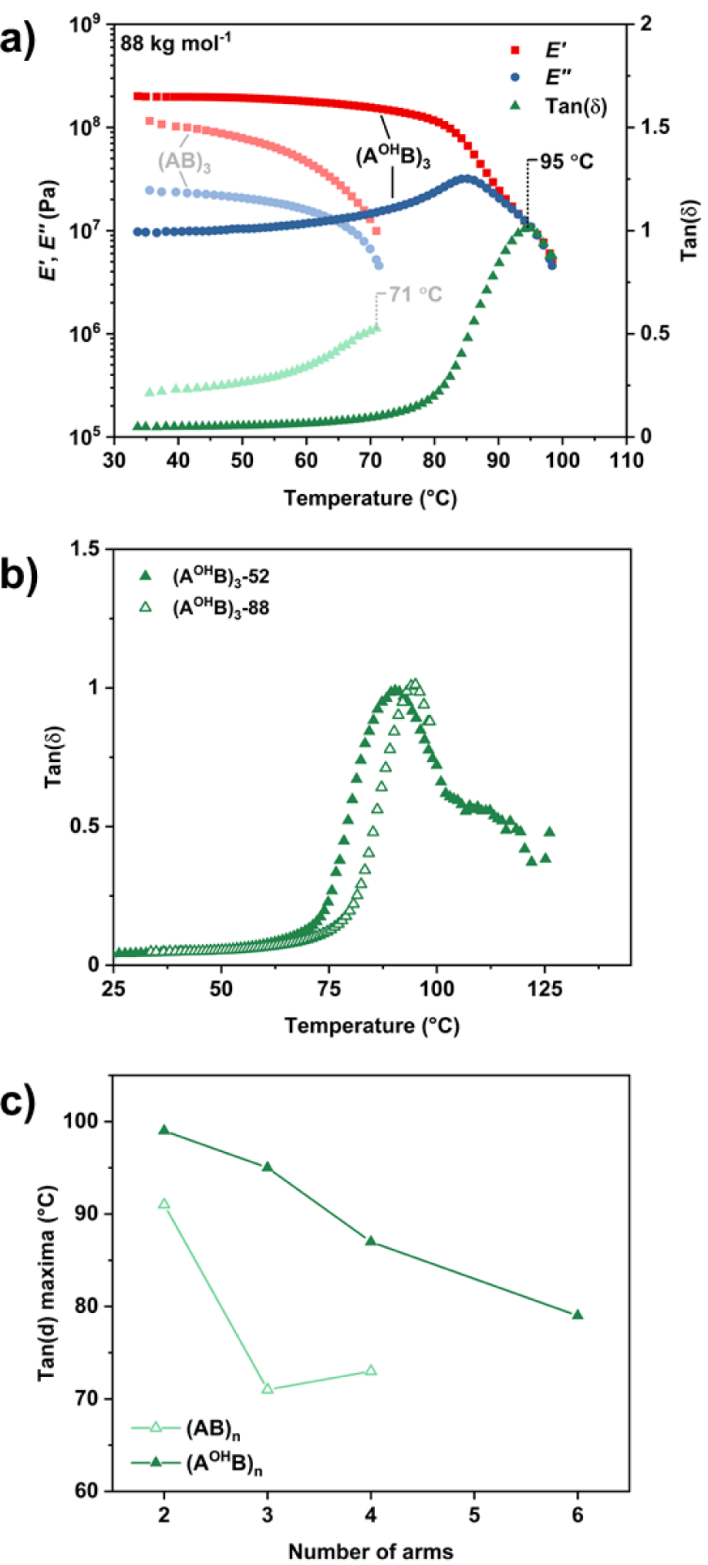
a) DMA thermal sweeps for high molar mass, nonmodified ((AB)_3_-88) and modified ((A^OH^B)_3_-88) 3-arm star block polymers. *T*_g_ values are highlighted as the peak maxima on the tan­(δ) curve. b) tan­(δ) versus temperature for 3-arm, modified star block polymers at high and low molar mass. c) Plot of the peak maxima in tan­(δ) from DMA against the number of arms for nonmodified and modified star block polymers.

The separation between the dynamic moduli was increased for all specimens after functionalization; in most cases, star samples showed a lower viscous component (decreased *E*″) and a higher elastic component (increased *E*′) vs linear analogues. This should correlate to a stiffer material with a lower degree of damping, as more energy is stored rather than dissipated.

Despite the difference in molar mass between the two 3-arm samples, only a slight increase in the temperature at which tan­(δ) peaked was observed for the higher molar mass sample and little difference in the degree of damping (Figure 4b). This was an interesting observation given the more significant contrast observed in the thermal transitions and mechanical properties of the two precursor samples. A decrease in the temperature for the maximum in tan­(δ) (i.e., upper *T*_g_) with increasing arm number is observed for the modified star polymers (Figure 4c). The greater difference between precursor and modified *T*_g_ for 3-arm and higher star samples compared with the linear analogue underscores the greater benefit of functionalization for the star architectures compared to the linear sample in enhancing microphase separation.

Tensile testing of the modified star block polymers was performed on specimens cut from solvent-cast films, and their mechanical properties were determined from the stress–strain profiles produced by uniaxial extension experiments (Table S5). All samples showed comparable values for Young’s modulus, which indicated similar stiffness in the elastic deformation region. Moreover, as the number of arms increased (*n* = 3, 4, 6), so did *E*_y_ compared to its respective precursor material, with the largest quantifiable improvement observed for (A^OH^B)_3_-52. Notably, functionalization of the linear analogue in (A^OH^B)_2_-63 appeared to show detrimental effects across all mechanical properties, whereas higher-arm samples were enhanced to varying degrees in terms of strength and stiffness.

The effect of the difference in molar mass on the mechanical performance of the two 3-arm samples was significantly less pronounced after functionalization (Figure 5a). Furthermore, a greater elongation at break was observed for (A^OH^B)_3_-52 resulting in a large increase in the material’s ductility compared with its precursor. In contrast, (A^OH^B)_3_-88 experienced a considerable reduction in its extensibility, suggesting that lower molar mass samples may be more influenced by postpolymerization modification.

**Figure 5:**
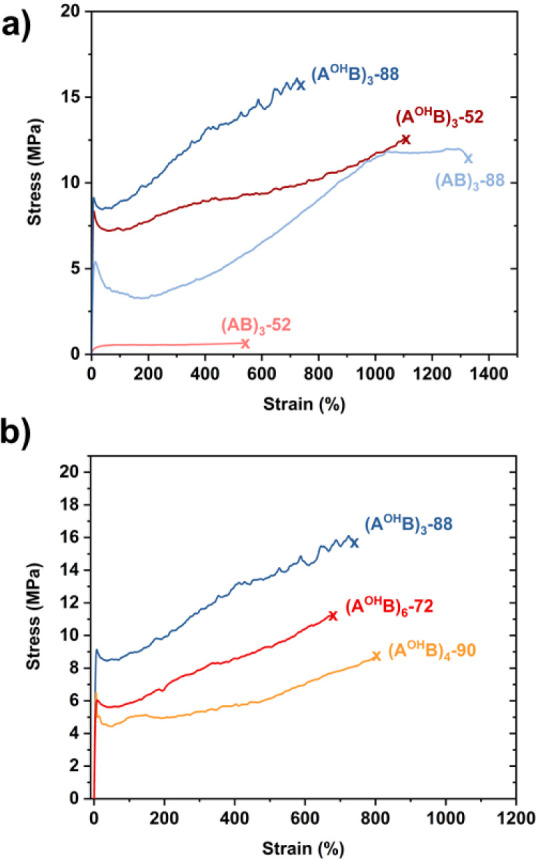
Representative stress–strain curves for a) nonmodified ((AB)_3_-#) 3-arm star block polymers and their modified analogues ((A^OH^B)_3_-#), and b) modified star block polymer samples, (A^OH^B)_*n*_, with *n* = 3, 4, and 6 arms. Failure points are marked with an “X”.

Broadly similar stress–strain profile shapes were observed for the higher molar mass samples with varying arm numbers (Figure 5b). The degrees of strain hardening illustrated in the representative curves and mean values for yield stress and strain were comparable across samples, regardless of arm number. Tensile stress and elongation at break values were also broadly similar across samples but with slightly greater variance. The data suggested that a beneficial limit in mechanical performance had been reached, possibly due to the greater block immiscibility after modification, resulting in all samples sharing a similar phase-separated microstructure and hence similar mechanical performance.

To investigate this notion, small-angle X-ray scattering (SAXS) experiments were conducted on nonmodified, (AB)_*n*_, and modified, (A^OH^B)_*n*_, thin film samples to determine the phase morphology. 1D SAXS patterns were plotted as scattering intensity (*I*(*q*)) against the scattering vector (*q*) and higher-order peak positions were indexed relative to the principal scattering peak (q*) (Table S6).

Nonmodified star block polymers displayed only the principal scattering peak, q*, with varying degrees of broadness, which indicated a weakly ordered or disordered system (Figures S36–S43). The broadest and least intense q* peak was observed for (AB)_3_-52 suggesting that it was disordered (Figure 6a). This correlated with the partial block miscibility identified by the broad, shifted *T*_g_ in the DSC, as well as the poor performance in mechanical testing.

**Figure 6:**
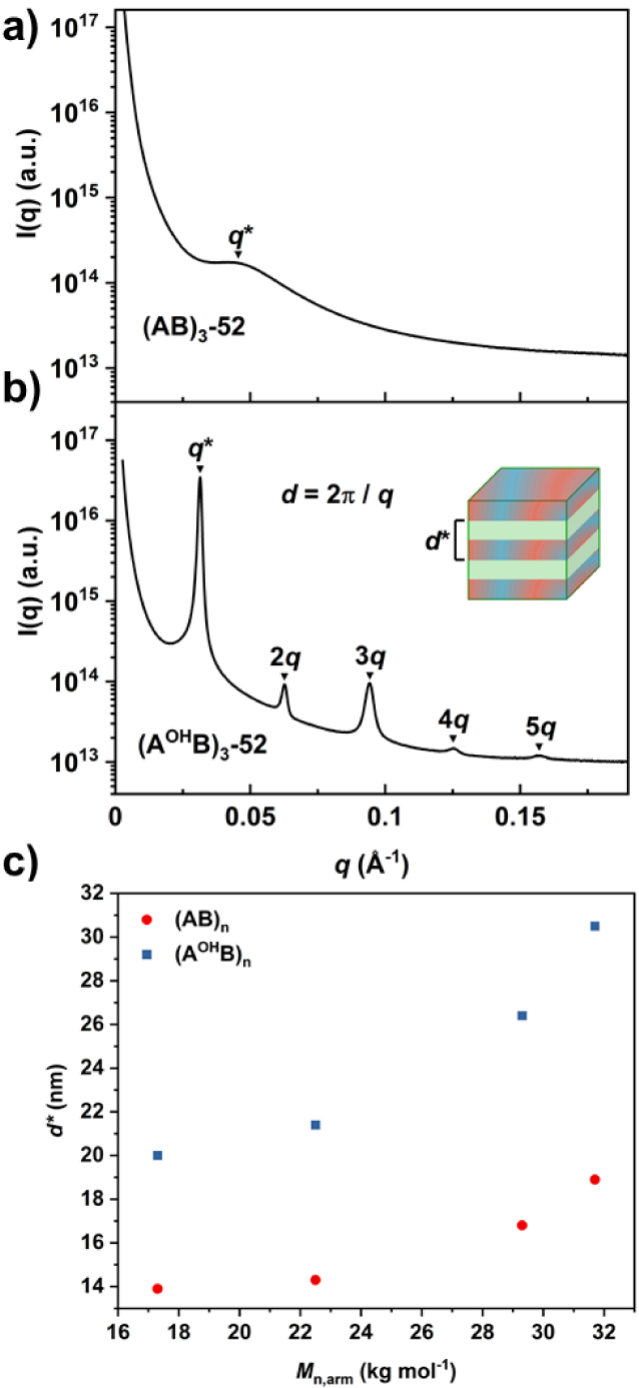
1D SAXS profiles for a) nonmodified ((AB)_3_-52) and b) modified ((A^OH^B)_3_-52) star block polymers. (A^OH^B)_3_-52 indexed for lamellar morphology, where d* represents domain periodicity. c) Plot of domain spacing (d*) against arm molar mass (*M*_n,arm_) for the series of nonmodified ((AB)_*n*_) and modified ((A^OH^B)_*n*_) star block polymers.

In all nonmodified samples, the absence of higher-order scattering peaks prevented the determination of phase morphology, but the position of q* allowed for the calculation of the principal domain spacing, d*. The largest domain spacing of 18.9 nm was observed for the linear analogue ((AB)_2_-63), while the low molar mass 3-arm star ((AB)_3_-52) showed the smallest spacing at 13.9 nm. However, it should be noted that this value is speculative given the broadness of q* and the likelihood of a disordered system (Figure 6a). Comparing the three higher molar mass samples, (AB)_2_-63, (AB)_3_-88, and (AB)_4_-90, revealed an inverse relationship between domain spacing and arm number—d* decreasing with increasing *n* (Table S5). A similar relationship was observed for (PDL-*b*-PLLA)_2–6_ star polymers of equal overall molar mass and relative hard/soft block ratio.^25^

In contrast, hydroxylated star block polymers showed sharper principal scattering peaks compared to their nonmodified counterparts, as well as higher-order scattering peaks at positions equivalent to integer values of q/q*, which indicated microstructures with long-range order (Figures S36–S43). The enhanced long-range ordering is attributed to the introduction of less favorable interactions between the two domains, an increase in the interaction parameter χ, and subsequently improved microphase separation. Indexing of the peak positions identified the morphology as lamellar (LAM), which is expected for symmetric star polymers with equal arm lengths at *f* ∼ 0.5 (Figure 6b).^41^

The principal domain spacing values calculated for the modified star polymers were larger and ranged from 20.0 to 30.5 nm. The same trend as with the nonmodified specimens was observed across the samples, with the linear polymer (A^OH^B)_2_-63 showing the largest domain spacing and (A^OH^B)_3_-52, the smallest. The inverse relationship between d* and *n* was again observed in the series of higher molar mass samples: (A^OH^B)_2_-63, (A^OH^B)_3_-88, and (A^OH^B)_4_-90 (Table S5). Furthermore, a plot of d* against *M*_n,arm_ for both nonmodified and modified samples highlights the overall shared trend of increased domain size with increasing arm molar mass (assumed to be proportional to arm size or length) (Figure 6c).

Interestingly, (A^OH^B)_3_-52 showed the largest number of scattering peaks (5), with sequentially fewer higher-order peaks observed for the polymer samples with increasing overall molar mass. In most samples, the higher-order reflection corresponding to 2*q* showed diminished intensity compared to the neighboring q* and 3*q* (Figure S39). Reduced intensity, or in some cases near absence, of 2*q* scattering is consistent with a structure factor extinction for compositionally symmetric, two-domain lamellar samples.^42,43^

## Conclusions

A series of multiarm (2-, 3-, 4-, 6-arm) star block polymers, each arm having polyester-*block*-polycarbonate structures, were successfully prepared using efficient and selective switchable catalysis, operating with one catalyst and one reactor. The relative hard (PvCHC) and soft (PDL) block content was fixed at 50 wt %, with overall molar masses ranging from 52 to 90 kg mol^–1^. The efficient introduction of hydroxyl groups to the PvCHC blocks enhanced microphase separation. Thermal, mechanical, and morphological analyses revealed that nonmodified star polymers exhibited some block miscibility, while hydroxyl-modified analogues demonstrated improved phase separation and a clearly defined lamellar morphology. Mechanical testing indicated that a higher arm molar mass correlated with increased strength and stiffness, while a greater number of arms generally reduced these properties. The unmodified star polymers displayed an absence of stress-whitening under a tensile load, suggesting a link between star architecture and fracture mechanics. Hydroxyl-modified star polymers exhibited consistent mechanical performance across all samples, likely due to the uniformity of their phase-separated microstructures. These findings underscore the significant impact of polymer architecture and functionalization on the mechanical and morphological properties of star block polymers.

## Supplementary Material



## Notes

Experimental and instrument details, polymer synthesis, characterization, and additional mechanical property measurements supplied (PDF)

## References

[1] Vidal F., van der Marel E. R., Kerr R. W. F., McElroy C., Schroeder N., Mitchell C., Rosetto G., Chen T. T. D., Bailey R. M., Hepburn C. (2024). Designing a circular carbon and plastics economy for
a sustainable future. Nature.

[2] Singh A., Rorrer N. A., Nicholson S. R., Erickson E., DesVeaux J. S., Avelino A. F. T., Lamers P., Bhatt A., Zhang Y., Avery G. (2021). Techno-economic,
life-cycle, and socioeconomic impact
analysis of enzymatic recycling of poly­(ethylene terephthalate). Joule.

[3] Zheng J., Suh S. (2019). Strategies to reduce the global carbon
footprint of plastics. Nat. Clim. Change.

[4] Haque F. M., Ishibashi J. S. A., Lidston C. A. L., Shao H., Bates F. S., Chang A. B., Coates G. W., Cramer C. J., Dauenhauer P. J., Dichtel W. R. (2022). Defining the Macromolecules of Tomorrow through
Synergistic Sustainable Polymer Research. Chem.
Rev..

[5] Zhu Y., Romain C., Williams C. K. (2016). Sustainable polymers from renewable
resources. Nature.

[6] Yang G.-W., Xie R., Zhang Y.-Y., Xu C.-K., Wu G.-P. (2024). Evolution of Copolymers
of Epoxides and CO2: Catalysts, Monomers, Architectures, and Applications. Chem. Rev..

[7] Hepburn C., Adlen E., Beddington J., Carter E. A., Fuss S., Mac Dowell N., Minx J. C., Smith P., Williams C. K. (2019). The technological
and economic prospects for CO2 utilization and removal. Nature.

[8] von
der Assen N., Jung J., Bardow A. (2013). Life-cycle assessment
of carbon dioxide capture and utilization: avoiding the pitfalls. Energy Environ. Sci..

[9] von
der Assen N., Bardow A. (2014). Life cycle assessment of polyols
for polyurethane production using CO2 as feedstock: insights from
an industrial case study. Green Chem..

[10] Artz J., Müller T. E., Thenert K., Kleinekorte J., Meys R., Sternberg A., Bardow A., Leitner W. (2018). Sustainable
Conversion of Carbon Dioxide: An Integrated Review of Catalysis and
Life Cycle Assessment. Chem. Rev..

[11] Darensbourg D. J., Wei S.-H., Yeung A. D., Ellis W. C. (2013). An Efficient Method
of Depolymerization of Poly­(cyclopentene carbonate) to Its Comonomers:
Cyclopentene Oxide and Carbon Dioxide. Macromolecules.

[12] McGuire T.
M., Deacy A. C., Buchard A., Williams C. K. (2022). Solid-State Chemical
Recycling of Polycarbonates to Epoxides and Carbon Dioxide Using a
Heterodinuclear Mg­(II)­Co­(II) Catalyst. J. Am.
Chem. Soc..

[13] Coates G. W., Getzler Y. D. Y. L. (2020). Chemical recycling to monomer for an ideal, circular
polymer economy. Nat. Rev. Mater..

[14] Poon K. C., Smith M. L., Williams C. K. (2024). Controlled
Carbon Dioxide Terpolymerizations
to Deliver Toughened yet Recyclable Thermoplastics. Macromolecules.

[15] Sulley G. S., Gregory G. L., Chen T. T. D., Peña Carrodeguas L., Trott G., Santmarti A., Lee K.-Y., Terrill N. J., Williams C. K. (2020). Switchable Catalysis
Improves the Properties of CO2-Derived
Polymers: Poly­(cyclohexene carbonate-b-ε-decalactone-b-cyclohexene
carbonate) Adhesives, Elastomers, and Toughened Plastics. J. Am. Chem. Soc..

[16] Scharfenberg M., Hilf J., Frey H. (2018). Functional
Polycarbonates from Carbon
Dioxide and Tailored Epoxide Monomers: Degradable Materials and Their
Application Potential. Adv. Funct. Mater..

[17] Deacy A. C., Gregory G. L., Sulley G. S., Chen T. T. D., Williams C. K. (2021). Sequence
Control from Mixtures: Switchable Polymerization Catalysis and Future
Materials Applications. J. Am. Chem. Soc..

[18] Yang G.-W., Wu G.-P. (2019). High-Efficiency Construction of CO2-Based Healable Thermoplastic
Elastomers via a Tandem Synthetic Strategy. ACS Sustainable Chem. Eng..

[19] Jia M., Zhang D., de Kort G. W., Wilsens C. H. R. M., Rastogi S., Hadjichristidis N., Gnanou Y., Feng X. (2020). All-Polycarbonate
Thermoplastic Elastomers Based on Triblock Copolymers Derived from
Triethylborane-Mediated Sequential Copolymerization of CO2 with Various
Epoxides. Macromolecules.

[20] Hassan M., Bhat G. A., Darensbourg D. J. (2024). Post-polymerization
functionalization
of aliphatic polycarbonates using click chemistry. Polym. Chem..

[21] Poon K. C., Gregory G. L., Sulley G. S., Vidal F., Williams C. K. (2023). Toughening
CO2-Derived Copolymer Elastomers Through Ionomer Networking. Adv. Mater..

[22] Scharfenberg M., Seiwert J., Scherger M., Preis J., Susewind M., Frey H. (2017). Multiarm Polycarbonate
Star Polymers with a Hyperbranched Polyether
Core from CO2 and Common Epoxides. Macromolecules.

[23] Ren J. M., McKenzie T. G., Fu Q., Wong E. H. H., Xu J., An Z., Shanmugam S., Davis T. P., Boyer C., Qiao G. G. (2016). Star Polymers. Chem. Rev..

[24] Shim J. S., Asthana S., Omura N., Kennedy J. P. (1998). Novel thermoplastic
elastomers. I. Synthesis and characterization of star-block copolymers
of PSt-b-PIB arms emanating from cyclosiloxane cores. J. Polym. Sci., Part A: polym. Chem..

[25] Lee S., Lee K., Jang J., Choung J. S., Choi W. J., Kim G.-J., Kim Y.-W., Shin J. (2017). Sustainable poly­(ε-decalactone)–poly­(l-lactide)
multiarm star copolymer architectures for thermoplastic elastomers
with fixed molar mass and block ratio. Polymer.

[26] Gruszka W., Garden J. A. (2021). Advances in heterometallic
ring-opening (co)­polymerisation
catalysis. Nat. Commun..

[27] Hilf J., Schulze P., Seiwert J., Frey H. (2014). Controlled
Synthesis
of Multi-Arm Star Polyether–Polycarbonate Polyols Based on
Propylene Oxide and CO2. Macromol. Rapid Commun..

[28] Liffland S., Hillmyer M. A. (2021). Enhanced Mechanical Properties of Aliphatic Polyester
Thermoplastic Elastomers through Star Block Architectures. Macromolecules.

[29] Liffland S., Kumler M., Hillmyer M. A. (2023). High Performance
Star Block Aliphatic
Polyester Thermoplastic Elastomers Using PDLA-b-PLLA Stereoblock Hard
Domains. ACS Macro Lett..

[30] Blankenship J. R., Levi A. E., Goldfeld D. J., Self J. L., Alizadeh N., Chen D., Fredrickson G. H., Bates C. M. (2022). Asymmetric Miktoarm
Star Polymers as Polyester Thermoplastic Elastomers. Macromolecules.

[31] Stößer T., Sulley G. S., Gregory G. L., Williams C. K. (2019). Easy access to oxygenated
block polymers via switchable catalysis. Nat.
Commun..

[32] Liu J., Jia M., Gnanou Y., Feng X. (2024). Heat-Resistant CO2-Based Polycarbonate
Thermoplastics. Macromolecules.

[33] Spyros A., Argyropoulos D. S., Marchessault R. H. (1997). A Study of Poly­(hydroxyalkanoate)­s
by Quantitative 31P NMR Spectroscopy: Molecular Weight and Chain Cleavage. Macromolecules.

[34] Rosetto G., Vidal F., McGuire T. M., Kerr R. W. F., Williams C. K. (2024). High Molar
Mass Polycarbonates as Closed-Loop Recyclable Thermoplastics. J. Am. Chem. Soc..

[35] Martello M. T., Schneiderman D. K., Hillmyer M. A. (2014). Synthesis and Melt Processing of
Sustainable Poly­(ε-decalactone)-block-Poly­(lactide) Multiblock
Thermoplastic Elastomers. ACS Sustainable Chem.
Eng..

[36] Shim J. S., Kennedy J. P. (1999). Novel thermoplastic elastomers. II. Properties of star-block
copolymers of PSt-b-PIB arms emanating from cyclosiloxane cores. J. Polym. Sci., Part A: polym. Chem..

[37] Kwee T., Taylor S. J., Mauritz K. A., Storey R. F. (2005). Morphology and mechanical
and dynamic mechanical properties of linear and star poly­(styrene-b-isobutylene-b-styrene)
block copolymers. Polymer.

[38] Burns A. B., Register R. A. (2016). Mechanical Properties
of Star Block Polymer Thermoplastic
Elastomers with Glassy and Crystalline End Blocks. Macromolecules.

[39] Blasco E., Sims M. B., Goldmann A. S., Sumerlin B. S., Barner-Kowollik C. (2017). Anniversary
Perspective: Polymer Functionalization. Macromolecules.

[40] Gregory G. L., Williams C. K. (2022). Exploiting Sodium Coordination in Alternating Monomer
Sequences to Toughen Degradable Block Polyester Thermoplastic Elastomers. Macromolecules.

[41] Matsen M. W., Schick M. (1994). Microphase Separation
in Starblock Copolymer Melts. Macromolecules.

[42] Epps T. H., Cochran E. W., Bailey T. S., Waletzko R. S., Hardy C. M., Bates F. S. (2004). Ordered Network Phases in Linear Poly­(isoprene-b-styrene-b-ethylene
oxide) Triblock Copolymers. Macromolecules.

[43] Pitet L. M., van Loon A. H. M., Kramer E. J., Hawker C. J., Meijer E. W. (2013). Nanostructured
Supramolecular Block Copolymers Based on Polydimethylsiloxane and
Polylactide. ACS Macro Lett..

