# Supplementary material for: (AB) n Star Block Polymers Derived from CO2: Influence of Architecture and Postpolymerization Modification
Source: Macromolecules. 2025 Oct 13;58(20):11291–301. doi: 10.1021/acs.macromol.5c01236 (PMC12574218; doi:10.1021/acs.macromol.5c01236)
Supplement: Supplementary file 1 [file ma5c01236_si_001.pdf]

Supporting Information

$(AB)_n$ Star Block Polymers Derived from CO_2: Influence of Architecture and Post-Modification

Gregory S. Sulley, Kam C. Poon, Georgina L. Gregory and Charlotte K. Williams*

Chemistry Research Laboratory, Department of Chemistry, University of Oxford, Oxford, OX1 3TA, U.K.

KEYWORDS: Carbon dioxide, thermoplastic, epoxide, lactone, polyester, polycarbonate, ring-opening polymerization, ring-opening copolymerization

G. S. Sulley, K. C. Poon, G. L. Gregory, Prof. C. K. Williams

Chemistry Research Laboratory, Department of Chemistry, University of Oxford, Oxford, OX1 3TA, U.K.

E-mail: charlotte.williams@chem.ox.ac.uk

Contents

General Methods 4

Materials 4

Analytical techniques 4

Experimental Procedures 6

Synthesis of heterodinuclear catalyst [LZnMg(C_6F_5)$_2$][4] 6

Representative Switch Polymerisation of ε-Decalactone, 4-Vinyl-1-cyclohexene-1,2-epoxide, and CO_2 for $(AB)_n$ Star Block Polymer Synthesis 7

$^{31}P\{^1H\}$ NMR Spectroscopy for Polymer End-group Titration 7

Representative Thiol-ene Post-polymerization Functionalization 7

Table S1. Multiarm star $(PDL)_n$ Characterisation Data 8

Figure S1. ^1H NMR spectra of purified 3- and 4-arm star poly(ε-decalactone). Highlighting signals for backbone, core, and end-group protons [400 MHz, $CDCl_3$]. 9

Figure S2. ^1H NMR spectrum of purified 3-arm star poly(*ε*-decalactone) $(PDL)_3$. [400 MHz, $CDCl_3$]. 10

Figure S3. ^1H NMR spectrum of purified 4-arm star poly(*ε*-decalactone) $(PDL)_4$. [400 MHz, $CDCl_3$]. 10

Figure S4. $^{31}P\{^1H\}$ NMR end-group analysis of $(PDL)_n$ star polymers. 11

Figure S5. SEC chromatographs of 3-arm (red) and 4-arm (blue) star poly(*ε*-decalactone) samples. 11

Figure S6. Representative ^1H NMR spectrum of a purified $(AB)_n$ star block polymer highlighting signals used for determination of relative block ratio [400 MHz, $CDCl_3$]. 12

Figure S7. ^1H NMR spectrum of purified 2-arm star block polymer $(AB)_2$-63. [400 MHz, $CDCl_3$]. 12

Figure S8. ^1H NMR spectrum of purified 3-arm star block polymer $(AB)_3$-52. [400 MHz, $CDCl_3$]. 13

Figure S9. ^1H NMR spectrum of purified 3-arm star block polymer $(AB)_3$-88. [400 MHz, $CDCl_3$]. 13

Figure S10. ^1H NMR spectrum of purified 4-arm star block polymer $(AB)_4$-90. [400 MHz, $CDCl_3$]. 14

Figure S11. ^1H NMR spectrum of purified 6-arm star block polymer $(AB)_6$-72. [400 MHz, $CDCl_3$]. 14

Figure S12. SEC chromatograph for star block polymer sample $(AB)_3$-88. Highlighted values for M_n and $Đ_M$ corresponding to the majority distribution (central peak) and the overall chromatogram. 15

Figure S13. SEC chromatographs of $(AB)_n$ star block polymers. 15

Table S2. (PvCHC-*b*-PDL)$_n$ [$(AB)_n$] Star Block Polymer Characterization Data 16

Figure S14. ^1H DOSY NMR spectrum (500 MHz, $CDCl_3$) of $(AB)_2$-63. 16

Figure S15. ^1H DOSY NMR spectrum (500 MHz, $CDCl_3$) of $(AB)_3$-52. 17

Figure S16. ^1H DOSY NMR spectrum (500 MHz, $CDCl_3$) of $(AB)_3$-88. 17

Figure S17. ^1H DOSY NMR spectrum (500 MHz, $CDCl_3$) of $(AB)_4$-90. 18

Figure S18. ^1H DOSY NMR spectrum (500 MHz, $CDCl_3$) of $(AB)_6$-72. 18

Figure S19. Viscometry SEC elution trace of $(AB)_n$ star block polymers and number average intrinsic viscosities. 19

Figure S20. $^{31}P\{^1H\}$ NMR spectra comparing $(PDL)_3$ and $(AB)_3$ (A = PvCHC, B = PDL) end-group titrations. 20

Figure S21. $^{31}P\{^1H\}$ NMR end-group analysis of $(AB)_n$-# star block polymers. 20

Table S3. Thermogravimetric Analysis of $(AB)_n$ Unmodified and $(A^{OH}B)_n$ Modified Star Block Polymers 21

Figure S22. TGA profile for $(AB)_n$ unmodified star block polymers. 21

Figure S23. DSC thermograms for 2-arm star block polymer samples $(AB)_2$-63 (non-modified) and $(A^{OH}B)_2$-63 (modified)..22

Figure S24. DSC thermograms for 3-arm star block polymer samples $(AB)_3$-52 (non-modified) and $(A^{OH}B)_3$-52 (modified)..22

Figure S25. DSC thermograms for 3-arm star block polymer samples (AB)3-88 (non-modified) and (A^{OH}B)3-88 (modified)..23

Figure S26. DSC thermograms for 4-arm star block polymer samples $(AB)_4$-90 (non-modified) and $(A^{OH}B)_4$-90 (modified)..23

Figure S27. DSC thermograms for 6-arm star block polymer samples $(AB)_6$-72 (non-modified) and $(A^{OH}B)_6$-72 (modified)..24

Figure S29. Digital photographs of sample $(AB)_3$-88: (a) Pristine and fractured test specimens before and after uniaxial extension, respectively; (b) Central region of a sample during uniaxial extension with absence of stress-whitening...26

Table S4. $(A^{OH}B)_n$ Modified Star Block Polymer Thermal Characterisation Data 26

Figure S30. TGA profile for $(A^{OH}B)_n$ modified star block polymers. 27

Figure S31. $^{31}P\{^1H\}$ NMR end-group analysis of virgin and thermally reprocessed (120 ˚C, 1 ton m^{-2}, 30 mins) $(A^{OH}B)_3$-88. 27

Figure S32. DMA temperature sweep profiles for star block polymer samples $(AB)_2$-63 (non-modified) and $(A^{OH}B)_2$-63 (modified)..28

Figure S33. DMA temperature sweep profiles for star block polymer samples $(AB)_4$-90 (non-modified) and $(A^{OH}B)_4$-90 (modified)..28

Figure S34. DMA temperature sweep profiles for star block polymer sample $(A^{OH}B)_3$-52 (modified). 29

Figure S35. DMA temperature sweep profiles for star block polymer sample $(A^{OH}B)_6$-72 (modified). 29

Table S5. $(A^{OH}B)_n$ Modified Star Block Polymer Mechanical Characterisation Data 30

Table S6. Small-Angle X-ray Scattering (SAXS) Data of $(A^{[OH]}B)_n$ Star Block Polymers 30

Figure S36. 1D SAXS profile for 2-arm star block polymer sample $(AB)_2$-63. 31

Figure S37. 1D SAXS profile for hydroxyl-modified 2-arm star block polymer sample $(A^{OH}B)_2$-63. 31

Figure S38. 1D SAXS profile for 3-arm star block polymer sample $(AB)_3$-52. 32

Figure S39. 1D SAXS profile for hydroxyl-modified 3-arm star block polymer sample $(A^{OH}B)_3$-52. 32

Figure S40. 1D SAXS profile for 3-arm star block polymer sample $(AB)_3$-88. 33

Figure S41. 1D SAXS profile for hydroxyl-modified 3-arm star block polymer sample $(A^{OH}B)_3$-88. 33

Figure S42. 1D SAXS profile for 4-arm star block polymer sample $(AB)_4$-90. 34

Figure S43. 1D SAXS profile for hydroxyl-modified 4-arm star block polymer sample $(A^{OH}B)_4$-90. 34

General Methods

Materials

The macrocylic ligand, H_2L, was synthesized following a previously reported procedure.[1] Magnesium bis(1,1,1,3,3,3-hexamethyldisilazan-2-ide) (97%) and bis(pentafluorophenyl)zinc (97%) were purchased from Sigma-Aldrich and used as received. Solvents used for synthesis and polymerization were collected from a solvent purification system (SPS), degassed with three freeze-pump-thaw cycles, and stored over 4 Å molecular sieves under an inert atmosphere. Cyclohexene oxide (98 %) (CHO) was purchased from Acros Organics, dried by stirring over CaH_2, followed by fractional distillation at 130 °C. It was stored under a nitrogen atmosphere *trans*-1,2-Cyclohexanediol (98%, Sigma-Aldrich) (CHD) was recrystallized from ethyl acetate, dried in vacuo, and stored under a nitrogen atmosphere. 1,1,1-Tris(hydroxymethyl)propane (97%, Sigma-Aldrich) (TMP) was recrystallized from acetone:diethyl ether (50:50), dried in vacuo, and stored under a nitrogen atmosphere. Pentaerythritol (99%, Sigma-Aldrich) (PER) was recrystallized from water, dried in vacuo at 90 °C, and stored under a nitrogen atmosphere. Dipentaerythritol (>99%, Sigma-Aldrich) (DPE) was recrystallized from water, dried in vacuo at 90 °C, and stored under a nitrogen atmosphere. *ε*-Decalactone (*ε*-DL) was dried over CaH_2, followed by fractional distillation at 70 °C under reduced pressure, and kept under a nitrogen atmosphere. Research-grade carbon dioxide was dried through a Drierite column and two additional drying columns (Micro Torr, Model number: MC1-804FV) in series before use.

Analytical techniques

Nuclear Magnetic Resonance (NMR) Spectroscopy. ^{1}H, COSY, HSQC, HMBC, $^{31}P\{^{1}H\}$, $^{19}F\{^{1}H\}$ NMR spectra were obtained using a Bruker AVIII HD 400 NMR spectrometer. $^{13}C\{^{1}H\}$ NMR spectra were obtained using a Bruker AVII 500 NMR spectrometer. Chemical shifts reported from low to high field in units of ppm. Abbreviations for commonly observed signal splitting: singlet = s, doublet = d, triplet = t, quartet = q, doublet doublet = dd, multiplet = m, broad = br. Values for coupling (J) reported in Hertz (Hz).

Size Exclusion Chromatography (SEC). SEC analysis was carried out on a Shimadzu LC-20AD instrument, equipped with a PSS SDV 5 µm precolumn and two PSS SDV 5 µm linear M columns in series and a refractive index (RI) detector. HPLC grade THF (inhibitor-free) was used as the eluent at 1.0 mL min^{-1} at 30 °C. Samples were dissolved in THF (5-10 mg mL^{-1}) and passed through 0.2 µm PTFE syringe filters (VWR International) prior to analysis. Monodisperse polystyrene standards were used for calibration (Agilent EasiVials, PS Medium 2 mL).

Differential Scanning Calorimetry (DSC). Thermal properties of polymers were measure using a DSC3+ (Mettler-Toledo Ltd) calibrated using zinc and indium standards. A sealed, empty crucible was used as a reference. Samples were heated from 25 °C to 120-150 °C, at a rate of 10 °C min^{-1}, under N_2 flow (100 mL min^{-1}), followed by a 5 minute isotherm, at 120-150 °C, to erase thermal history. Samples were subsequently cooled to -90 °C, as a rate of 10 °C min^{-1}, and kept at -90 °C for a further 5 minutes, followed by a heating-cooling cycle procedure from -90 °C to 120-150 °C, at a rate of 10 °C min^{-1}. Each sample was analysed over two full heating-cooling cycles. Glass transition temperatures (T_g) are reported as the midpoint of the transition taken from the second heating cycle.

Thermogravimetric Analysis (TGA). Thermal decomposition of polymer samples was measured using a TGA/DSC 1 system (Mettler-Toledo Ltd). Samples were heated in an open, ceramic, crucible from 25 °C to 500 °C, at a rate of 5 °C min^{-1}, under N_2 flow (100 cm^3 min^{-1}).

Dynamic Mechanical [Thermal] Analysis (DM[T]A). Viscoelastic properties of free-stranding polymer films were analysed using a RSA-G2 Solids Analyser (TA Instruments). Specimens of uniform width were cut from a solvent cast film using a Zwick ZCP020 cutting press. Samples were heated from -70 °C to 40 °C and 35 °C to 80-130 °C at a rate of 5 °C min^{-1} for low and high temperature sweeps, respectively. The frequency was set to 1 Hz, the strain amplitude to 0.1%, and a 0.1 N pre-load was applied. Glass transition temperatures (T_g) are reported, where possible, as the peak maxima in tan(δ). Oscillatory rheology of softer specimens was performed on an Anton Paar Physica MCR301 rheometer equipped with 25 mm parallel plate (PP25) geometries. Frequency sweep analysis was conducted at 25 °C with γ = 0.5%, and a logarithmic frequency ramp between 0.01 and 30 Hz. The following parameters were used in other testing methods: γ = 0.5%, ω = 1 Hz.

Tensile Mechanical Analysis. Polymer specimens were analysed using an EZ-LX Universal Testing Instrument (Shimadzu). Dumbbell-shaped specimens were cut using a Zwick ZCP020 cutting press equipped with a cutting die for ISO 527-2 type 5B. Uniaxial extension experiments (10 mm min^{-1} cross-head speed) were run according to ISO 527. Cyclical hysteresis experiments (10 mm min^{-1} cross-head speed) were conducted from 0-200% strain over 10 cycles. Values for mechanical characteristics (e.g., Young's modulus, stress at break etc.) were averaged over at least 5 specimens.

Small-Angle X-ray Scattering (SAXS). Synchrotron SAXS analysis of block polymer films was carried out at beamline I22, located at Diamond Light Source, Harwell, United Kingdom. Samples were mounted either between two pieces of Kapton®, Scotch tape, or free in a solids sample grid. A monochromatic X-ray radiation (λ = 0.1 nm) and 2D SAXS detector (Pilatus P3-2M, DECTRIS Ltd) were used for experiments. 2D scattering patterns were reduced to 1D using Dawn software developed at the Diamond Light Source.[2, 3]

Experimental Procedures

Synthesis of heterodinuclear catalyst [LZnMg(C_6F_5)$_2$][4]

Under inert conditions, macrocyclic diphenol tetraamine-based pro-ligand (H_2L) (0.5 g, 0.90 mmol) and [Mg{N[Si(CH_3)$_3$]$_2$}$_2$] (0.31 g, 0.90 mmol) were stirred in anhydrous THF (10 mL) at 25 °C for 1 hour. A solution of Zn(C_6F_5)$_2$ (0.36 g, 0.90 mmol), in anhydrous THF (5 mL), was then added dropwise to the reaction solution which afforded an orange solution which was stirred overnight at 25 °C. The solvent was removed *in vacuo* to yield a pale orange solid. The product was isolated by washing with cold (-30 °C) THF (5 mL) and pentane (2 x 5 mL) followed by centrifugation. The off-white powder was dried *in vacuo* overnight at 25 °C (0.62 g, 70% yield).

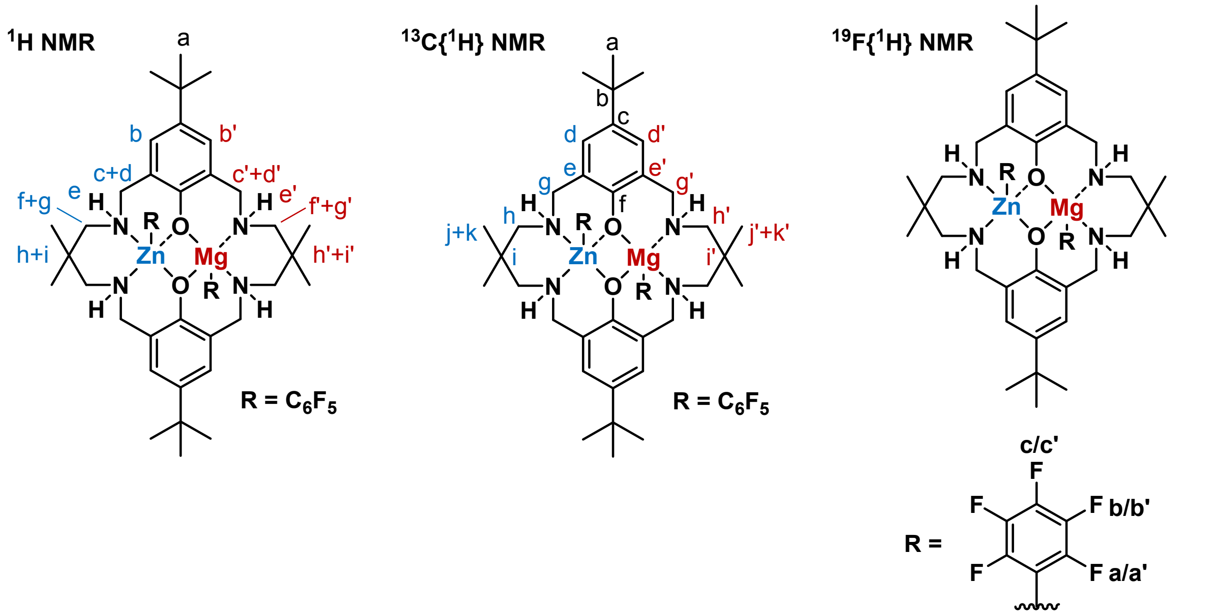

^{1}H NMR (400 MHz, $CDCl_3$, 298 K) δ(ppm): 6.81 (d, 2H, *J* = 3, b'), 6.76 (d, 2H, *J* = 3, b), 4.37 (m, 2H, c'), 4.30 (m, 2H, c), 3.40 (d, 2H, *J* = 14, d), 3.26 (d, 2H, *J* = 13, d'), 3.08 (m, 2H, f'), 3.01 (m, 2H, f), 2.78-2.63 (m, 6H, g+g'+e), 2.12-2.02 (m, 2H, e'), 1.27 (s, 3H, h), 1.20 (s, 18H, a), 1.17 (s, 3H, h'), 1.06-1.02 (m, 6H, i+i'). ^{13}C{^{1}H} (125 MHz, $CDCl_3$, 298 K) δ(ppm): 159.2 (f), 137.2 (c), 126.3 (d or d'), 126.3 (d' or d), 124.5 (e'), 122.3 (e), 62.6 (h), 61.7 (h'), 57.8 (g), 55.9 (g'), 34.2 (i or i'), 33.9 (i or i'), 33.5 (b), 31.5 (a), 28.3 (j+j' or k+k'), 28.3 (j+j' or k+k'), 20.3 (j+j' or k+k'), 20.1 (j+j' or k+k'). ^{19}F{^{1}H} (376 MHz, $CDCl_3$, 298 K) δ(ppm): -111.2 (m, 2F, *o*-C_6F_5), -116.2 (br, 2F, *o*-C_6F_5), -157.8 (m, 1F, *p*-C_6F_5), -159.5 (m, 1F, *p*-C_6F_5), -161.2 (m, 2F, *m*-C_6F_5), -161.8 (m, 2F, *m*-C_6F_5). Anal. Calc. for $C_{46}H_{54}F_{10}MgN_4O_2Zn$ (%): C 56.69, H 5.58, N 5.75. Found: C 56.55, H 5.61, N 5.68.

N.B. Assignment of signals as being on the zinc or magnesium "side" of the macrocycle and co-ligands was achieved using 2D NMR spectroscopic techniques (^{1}H and ^{19}F{^{1}H} COSY, ^{1}H-^{13}C HSQC and HMBC), and by comparison to spectra of a previously synthesised homodinuclear zinc complex.[5]

Representative Switch Polymerisation of ε-Decalactone, 4-Vinyl-1-cyclohexene-1,2-epoxide, and CO_2 for $(AB)_n$ Star Block Polymer Synthesis

Under anaerobic conditions, $[LZnMg(C_6F_5)_2]$ (20 mg, 0.02 mmol), the chosen chain-transfer agent (CHD, TMP, PER, DPE) (0.08 mmol), ε-DL (3.3 mL, 18.8 mmol), and toluene (7.8 mL) were added to a flame-dried vial. The vial was stirred at 80 °C for 1 hour, after which it was cooled and an aliquot was taken to determine conversion of ε-DL to PDL by ^1H NMR spectroscopy. To the remaining solution in the vial was added vCHO (2.5 mL, 19.0 mmol), and the entire contents transferred to a stainless steel Parr pressure vessel. The vessel was then pressurised with CO_2 to 20 bar and stirred at 80 °C for 20 hours, before being cooled to 35 °C and the remaining CO_2 pressure vented. A sample of the crude polymer was removed for analysis by ^1H NMR spectroscopy. SEC analysis was performed on a crude sample after removal of residual solvent. The resulting polymer was isolated by precipitation twice from a solution of THF (< 1 g mL^{-1}) into methanol (200 mL), and dried *in vacuo* to a constant weight.

$^{31}P\{^1H\}$ NMR Spectroscopy for Polymer End-group Titration

A reported procedure for the analysis of hydroxyl end-groups was followed.[6] The polymer sample (20 mg) and stock solution (40 µL), in $CDCl_3$ (0.5 mL), were mixed in an NMR tube. Excess 2-chloro-4,4,5,5-tetramethyldioxaphospholane (40 µL) was then added to the NMR tube and shaken to combine. The mixture was allowed to react for 6 h before analysis by $^{31}P\{^1H\}$ NMR experiment. The contents of the stock solution were bisphenol A (400 mg), $Cr(acac)_3$ (5.5 mg), and pyridine (10 mL).[7]

Representative Thiol-ene Post-polymerization Functionalization

To a flame-dried vial, under anaerobic conditions, was added the sample of polymer (1.0 g, 2.97 mmol of PvCHC repeat unit) in THF (5 mL). Once fully dissolved, 2,2-dimethoxyphenylacetophenone (DMPA) (305 mg, 1.20 mmol) and 2-mercaptoethanol (ME) (0.83 mL, 11.9 mmol) were added to the solution. The vial was sealed securely and stirred under UV irradiation (λ = 365 nm) for 2 hours, at which point the reaction was quenched by exposure to air. The crude material was isolated by precipitation twice from a solution of THF into diethyl ether (200 mL), filtered and washed with further diethyl ether (2 x 10 mL), and then dried *in vacuo* to a constant weight.

Scheme S1. Synthesis of $(PDL)_n$ star polymers.

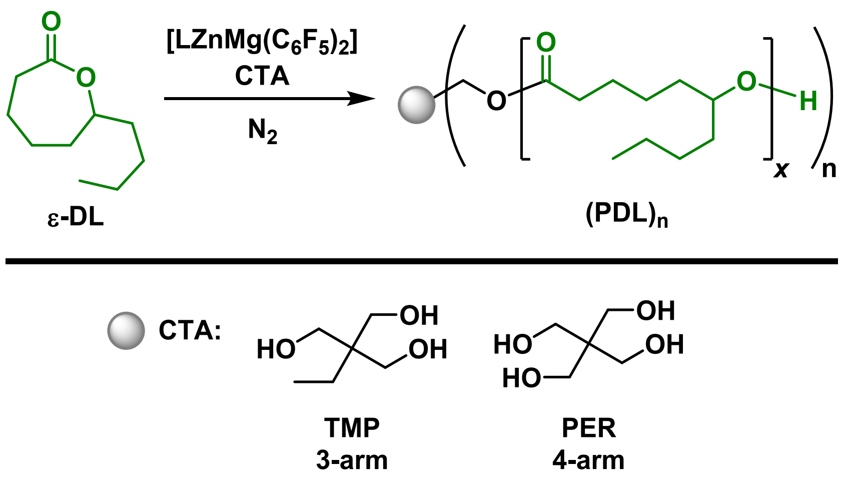

Table S1. Multiarm star $(PDL)_n$ Characterisation Data

CTA[a]	n_{OH}[b]	Time (min)	Conversion (%)[c]	DP_{total}[d]	$M_{n,NMR}$[e] (kg mol^{-1})	$M_{n,SEC}$[f] (kg mol^{-1})	$Đ_M$[g]
TMP	3	15	44	27	4.6	7.6	1.09
PER	4	30	95	62	10.6	13.9	1.19

[a]Reaction conditions: [**2**]:[CTA]:[ε-DL] = 1:4:200, 80 °C, toluene ($[\varepsilon\text{-DL}]_0$ = 1.7 M); TMP = 1,1,1-Tris(hydroxymethyl)propane, PER = Pentaerythritol. [b]Number of hydroxide functional groups on the CTA, i.e. theoretical maximum number of star arms. [c]Determined from ^{1}H NMR spectroscopy of crude polymer samples by comparing relative integrals of *ε*-DL (4.21 ppm) and PDL (4.85 ppm). [d]Total degree of polymerisation; calculated from ^{1}H NMR spectra of purified polymer samples by comparing integrals for PDL backbone (4.85 ppm) and end-group (3.58 ppm). [e]Determined from ^{1}H NMR spectroscopy, (DP_{total} * 170.25). [f]Determined from SEC analysis in THF, calibrated using narrow polystyrene standards. [g]M_w/M_n.

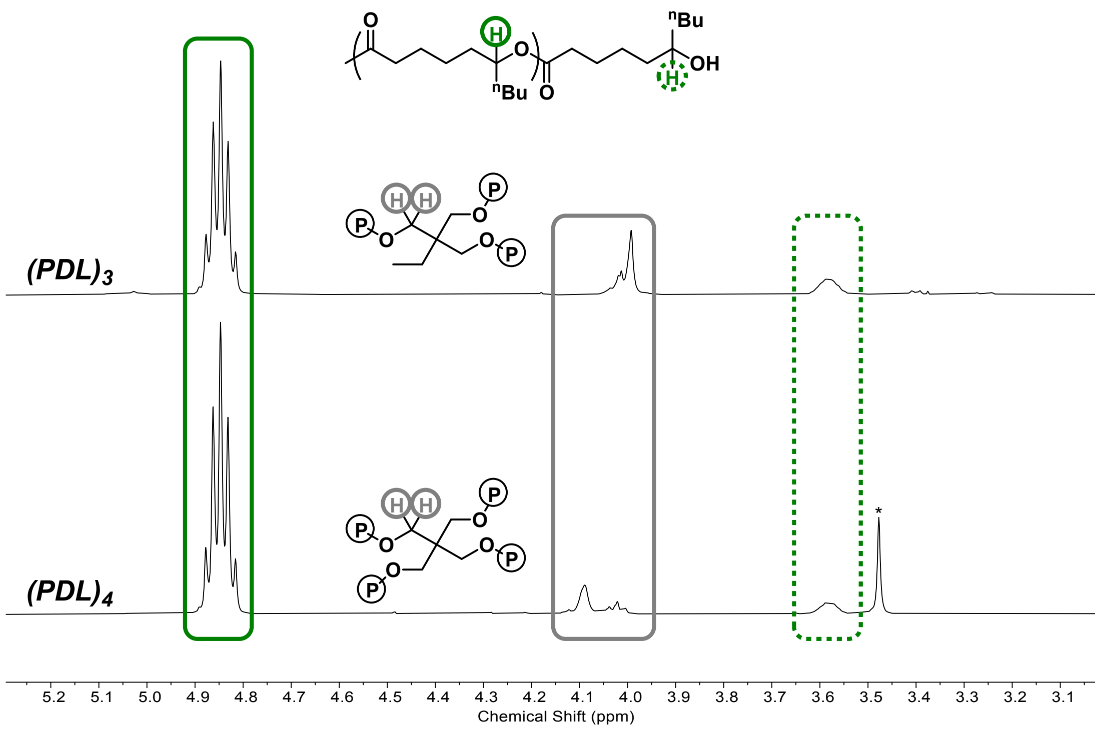

Figure S1. ^{1}H NMR spectra of purified 3- and 4-arm star poly(ε-decalactone). Highlighting signals for backbone, core, and end-group protons [400 MHz, $CDCl_3$].

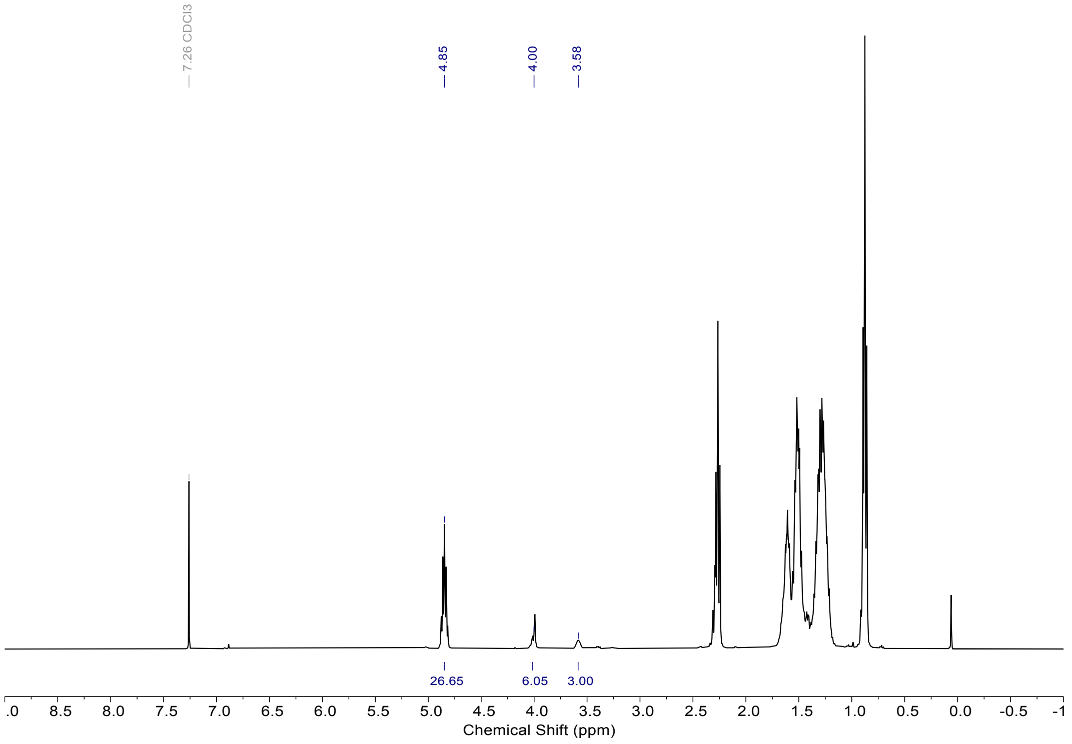

Figure S2. ^{1}H NMR spectrum of purified 3-arm star poly(*ε*-decalactone) $(PDL)_3$. [400 MHz, $CDCl_3$].

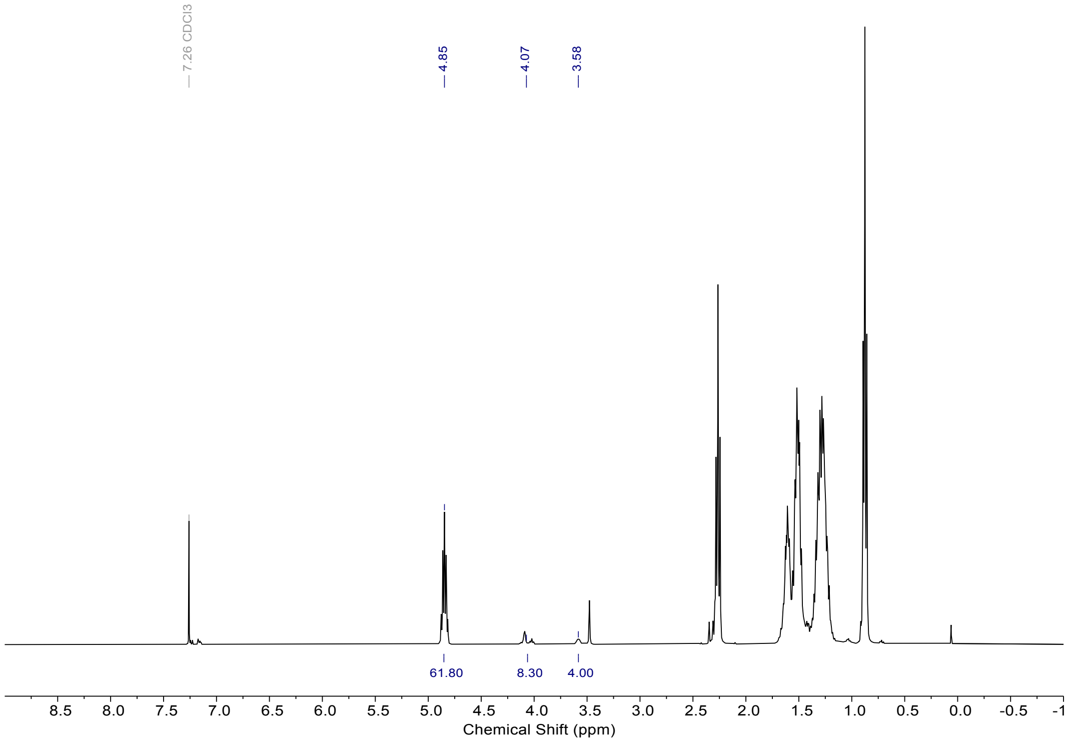

Figure S3. ^{1}H NMR spectrum of purified 4-arm star poly(*ε*-decalactone) $(PDL)_4$. [400 MHz, $CDCl_3$].

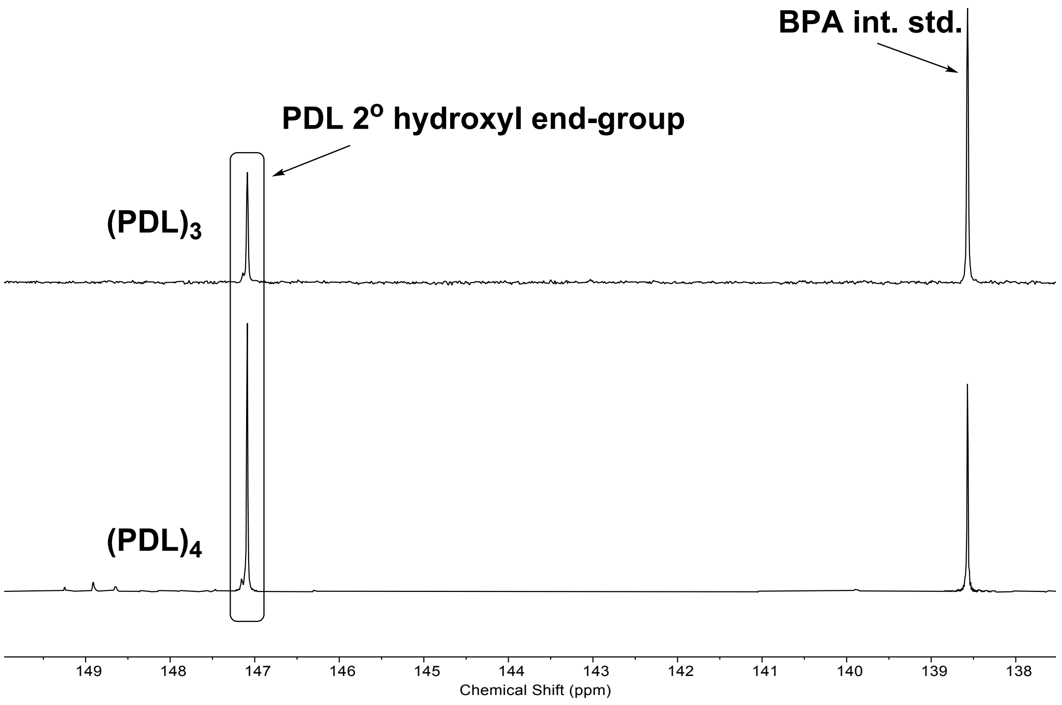

Figure S4. $^{31}P\{^1H\}$ NMR end-group analysis of $(PDL)_n$ star polymers.

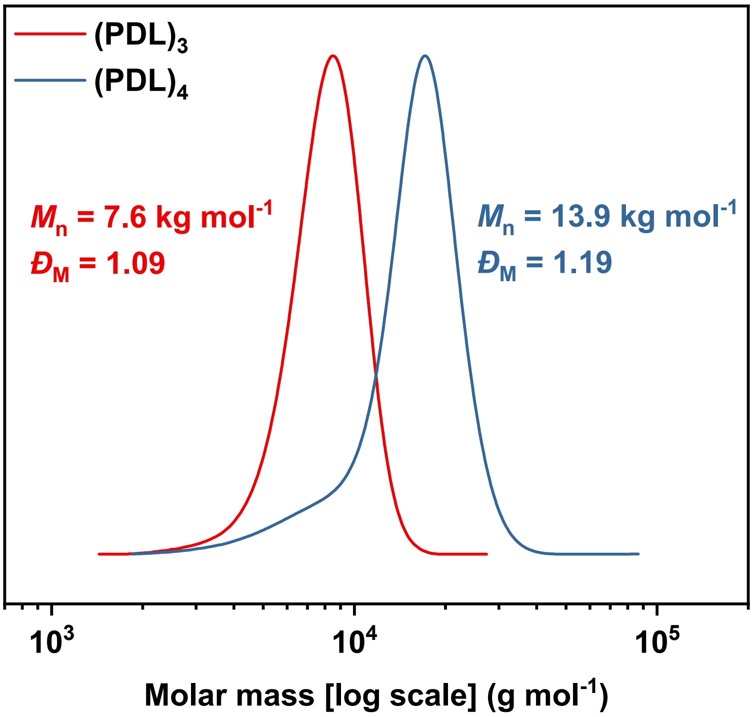

Figure S5. SEC chromatographs of 3-arm (red) and 4-arm (blue) star poly(ε-decalactone) samples.

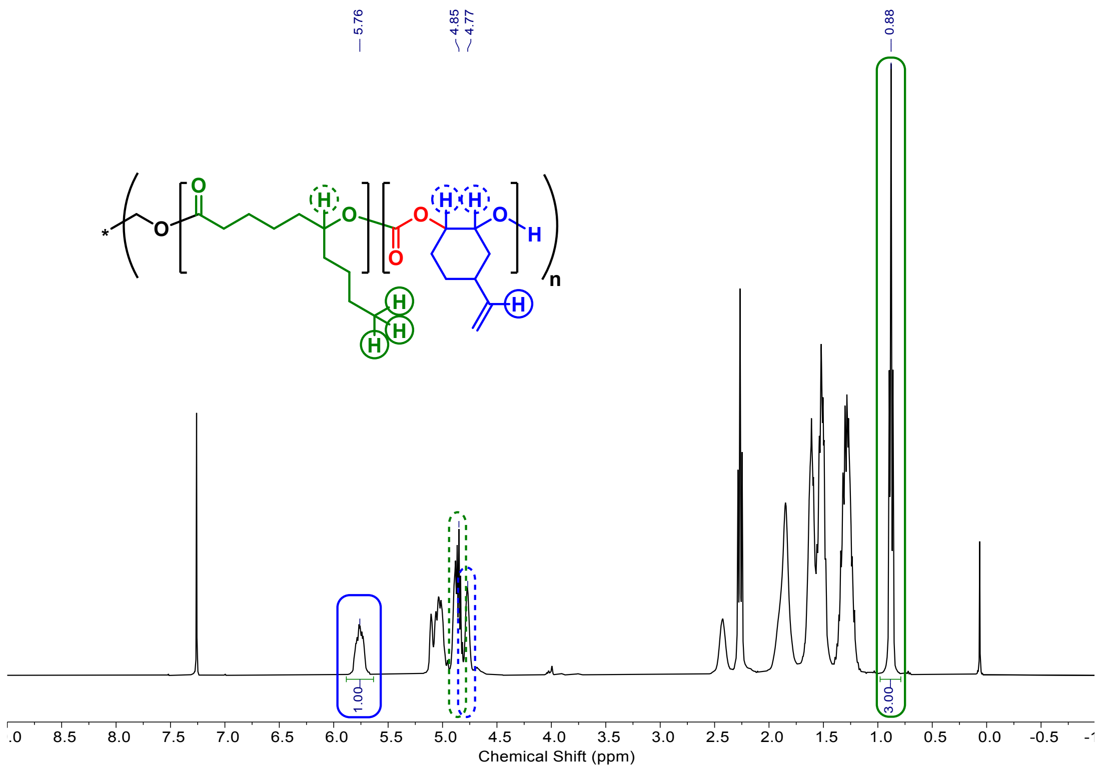

Figure S6. Representative ^{1}H NMR spectrum of a purified $(AB)_n$ star block polymer highlighting signals used for determination of relative block ratio [400 MHz, $CDCl_3$].

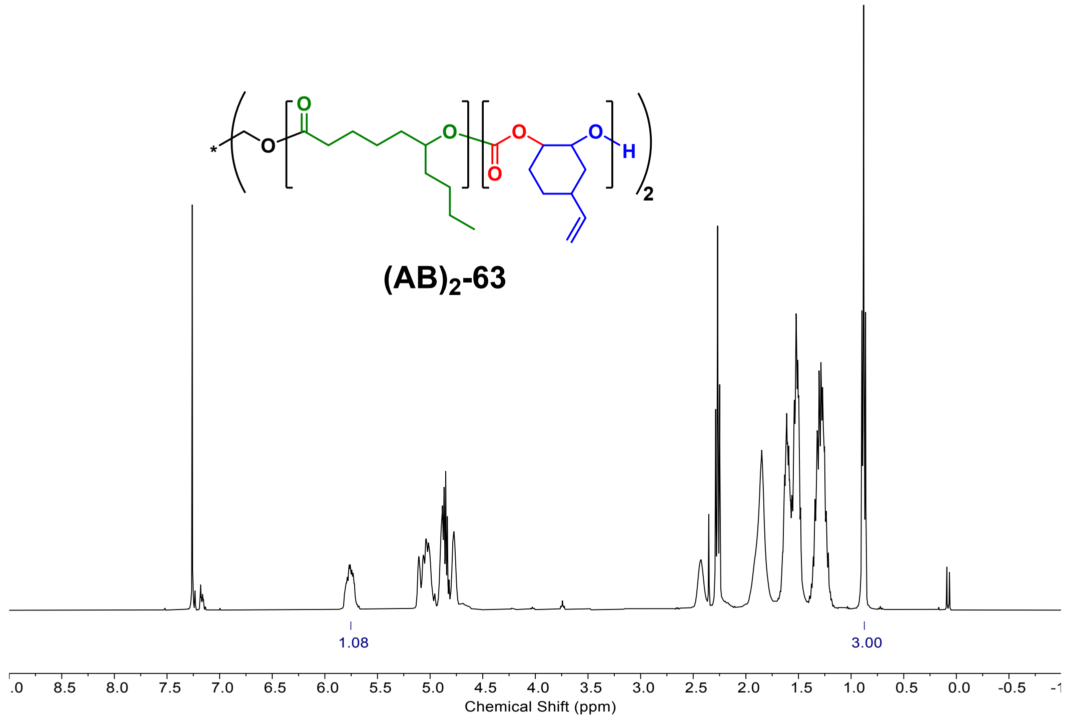

Figure S71. ^{1}H NMR spectrum of purified 2-arm star block polymer $(AB)_2$-63. [400 MHz, $CDCl_3$].

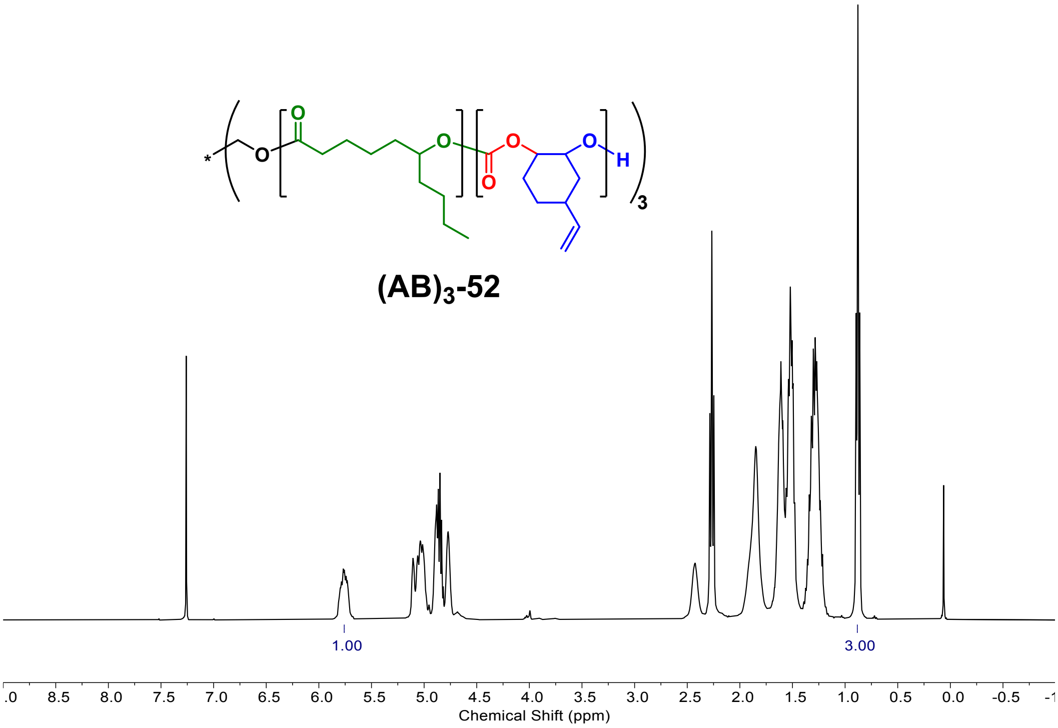

Figure S8. ^{1}H NMR spectrum of purified 3-arm star block polymer (AB)$_3$-52. [400 MHz, $CDCl_3$].

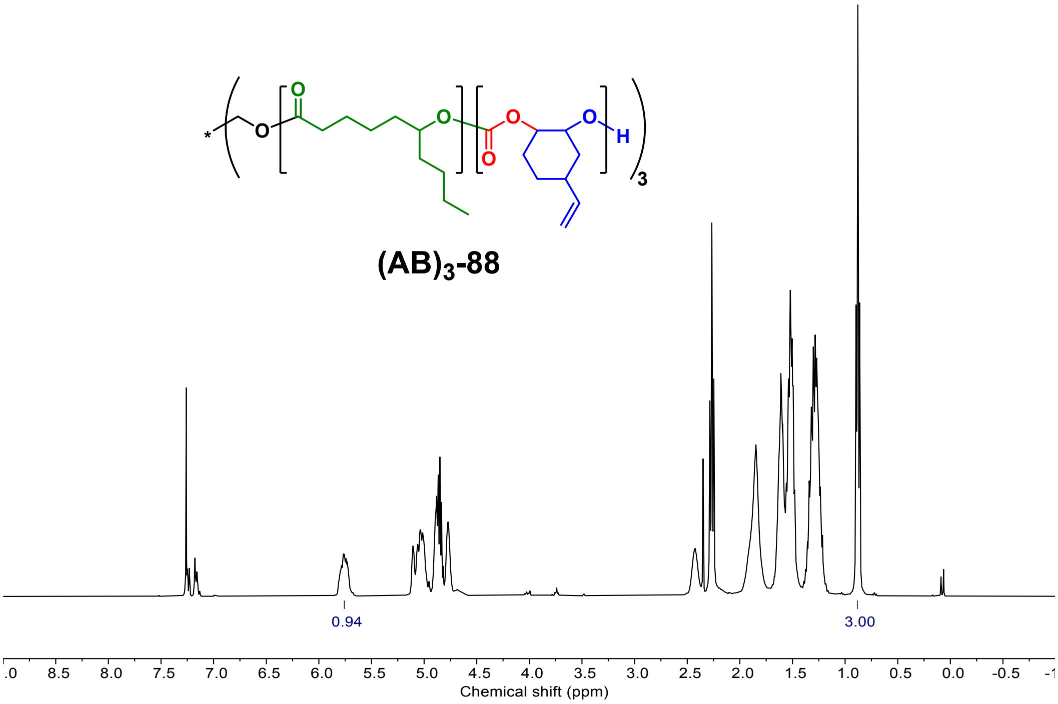

Figure S9. ^{1}H NMR spectrum of purified 3-arm star block polymer (AB)$_3$-88. [400 MHz, $CDCl_3$].

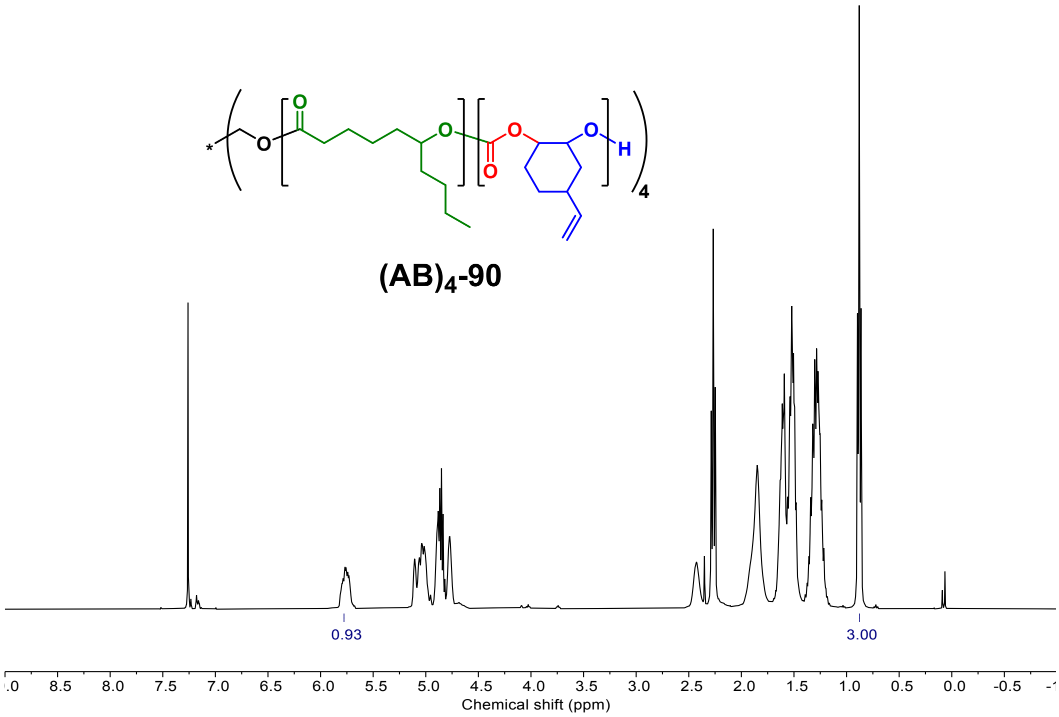

Figure S10. ^{1}H NMR spectrum of purified 4-arm star block polymer (AB)$_4$-90. [400 MHz, $CDCl_3$].

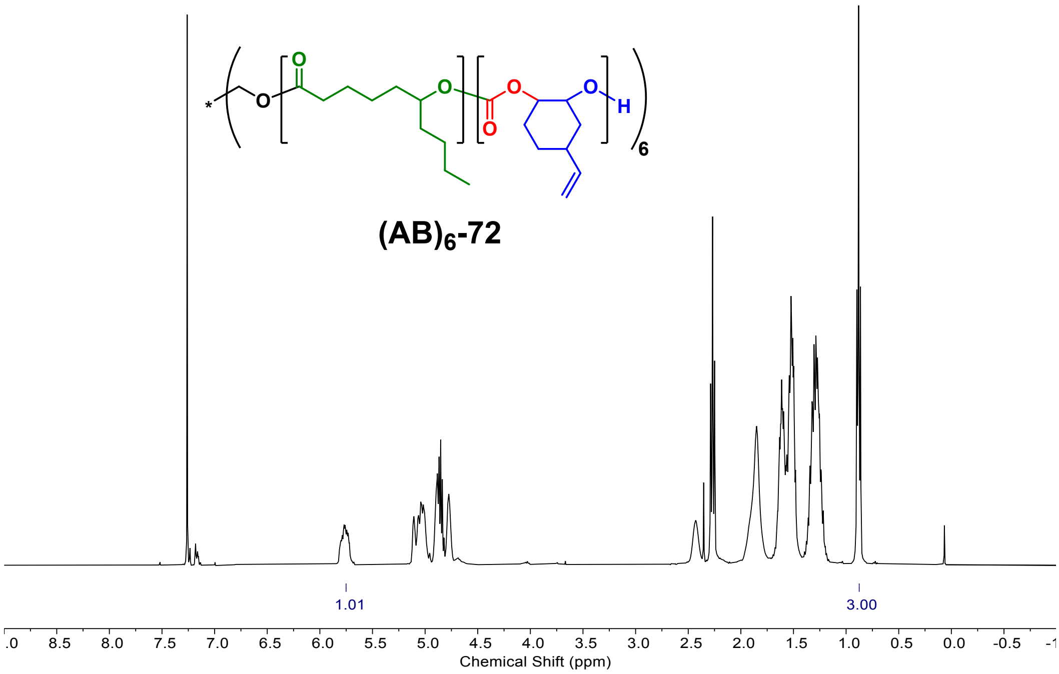

Figure S11. ^{1}H NMR spectrum of purified 6-arm star block polymer (AB)$_6$-72. [400 MHz, $CDCl_3$].

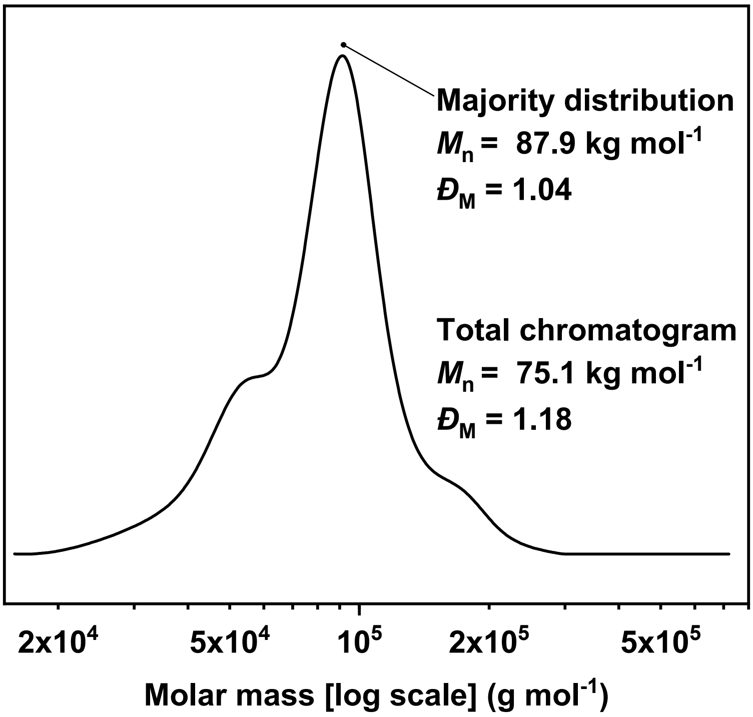

Figure S12. SEC chromatograph for star block polymer sample $(AB)_3$-88. Highlighted values for M_n and $Đ_M$ corresponding to the majority distribution (central peak) and the overall chromatogram.

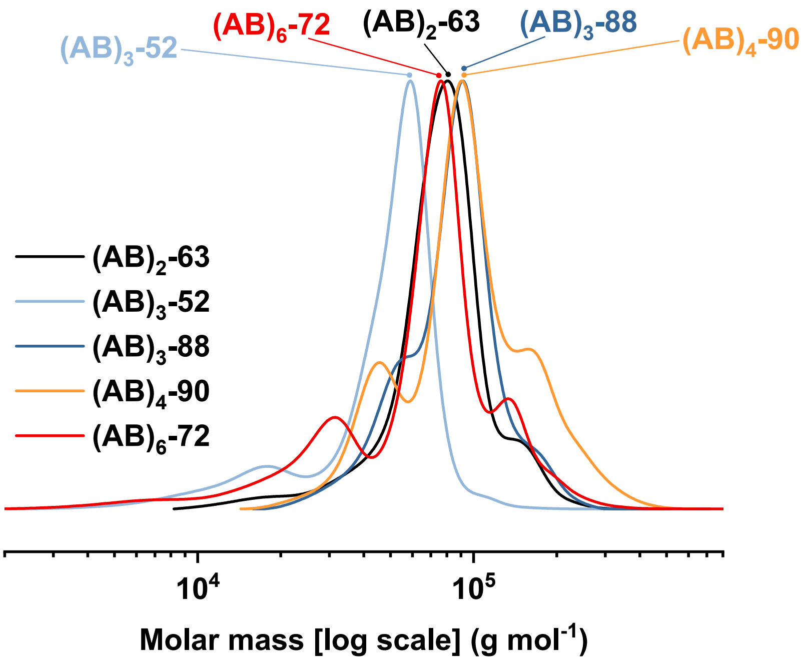

Figure S13. SEC chromatographs of $(AB)_n$ star block polymers.

Table S2. (PvCHC-*b*-PDL)$_n$ [(AB)$_n$] Star Block Polymer Characterization Data

Entry	Polymer[a]	n_{arms}[b]	$M_{n,SEC}$[c] (kg mol^{-1}) [$Đ_M$] Total[d]	$M_{n,SEC}$[c] (kg mol^{-1}) [$Đ_M$] Split[e]
1	(AB)$_2$-63	2	66.3 [1.22]	160 [1.02]<br>**63.3 [1.18]**
2	(AB)$_3$-52	3	36.0 [1.40]	**51.9 [1.08]**<br>12.4 [1.20]
3	(AB)$_3$-88	3	75.1 [1.18]	172 [1.02]<br>**87.9 [1.04]**<br>43.2 [1.06]
4	(AB)$_4$-90	4	79.9 [1.34]	192 [1.06]<br>**90.0 [1.05]**<br>40.4 [1.05]
5	(AB)$_6$-72	6	63.4 [1.34]	156 [1.07]<br>**71.9 [1.04]**<br>27.2 [1.08]

[a](AB)n-#; where A = PvCHC, B = PDL, n = number of arms, # = molar mass, major distribution (SEC). [b]Number of arms based on full functionalization from CTA. [c]Molar mass determined from SEC, $Đ_M$=M_w/M_n. [d]Molar mass data for entire chromatogram without peak deconvolution. [e]Molar mass data for constituent chromatogram peaks (bold indicates majority distribution), deconvoluted using Shimadzu GPC Postrun software.

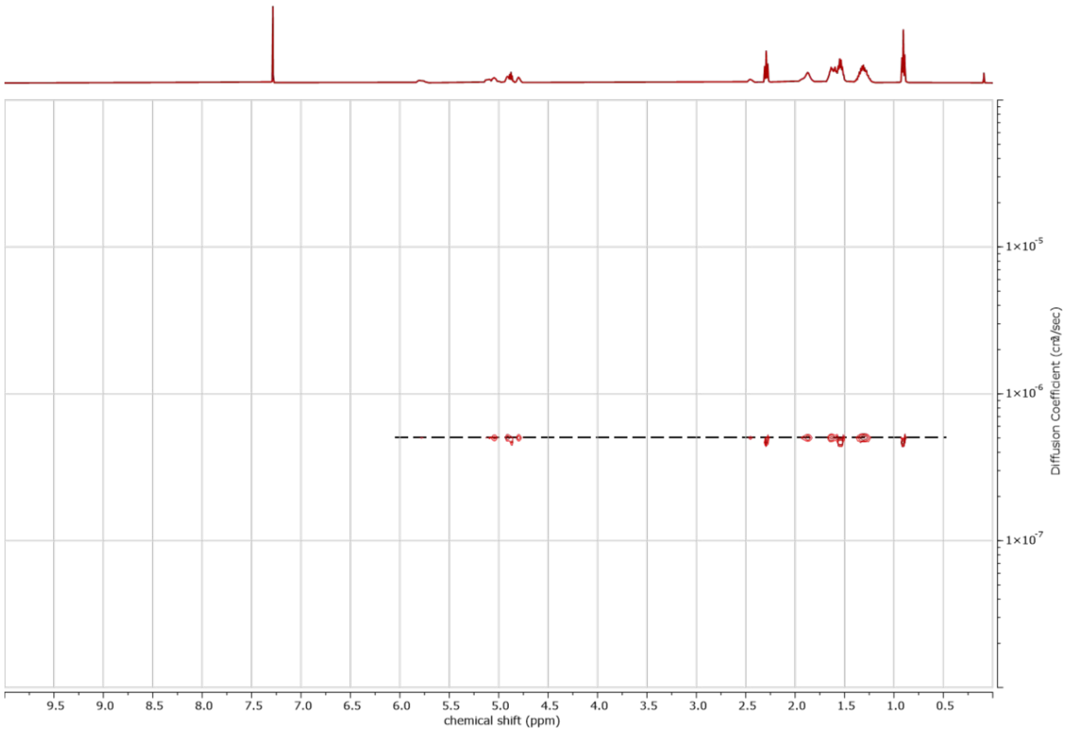

Figure S14. ^{1}H DOSY NMR spectrum (500 MHz, $CDCl_3$) of (AB)$_2$-63.

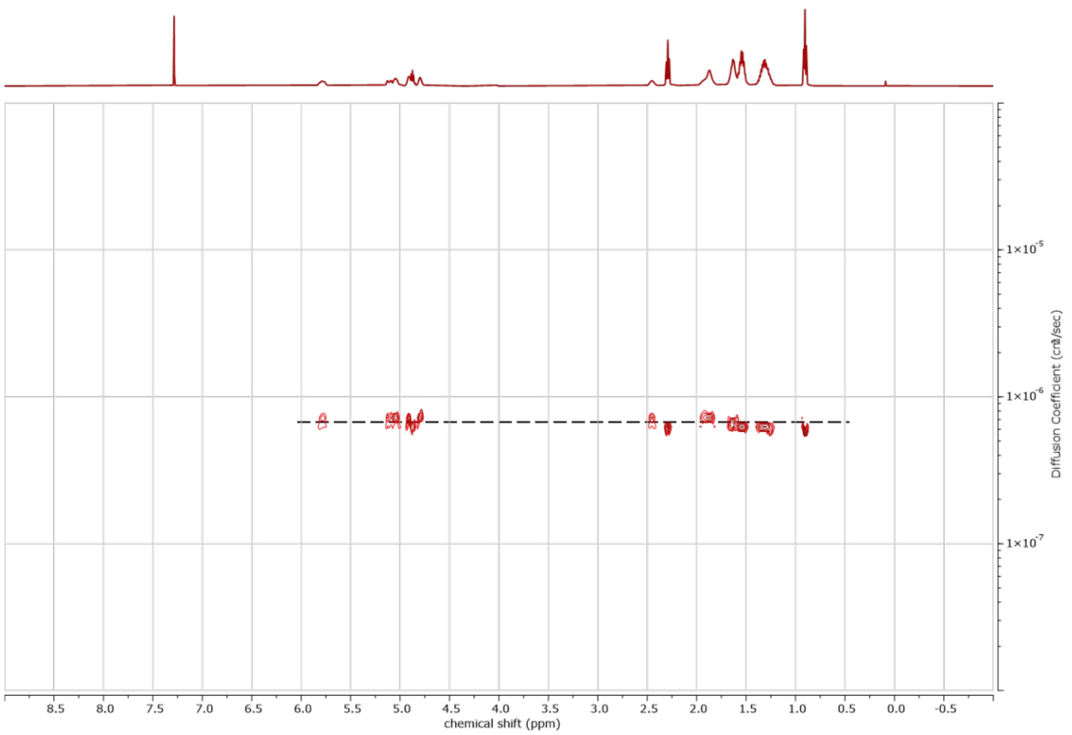

Figure S15. ^{1}H DOSY NMR spectrum (500 MHz, $CDCl_3$) of $(AB)_3$-52.

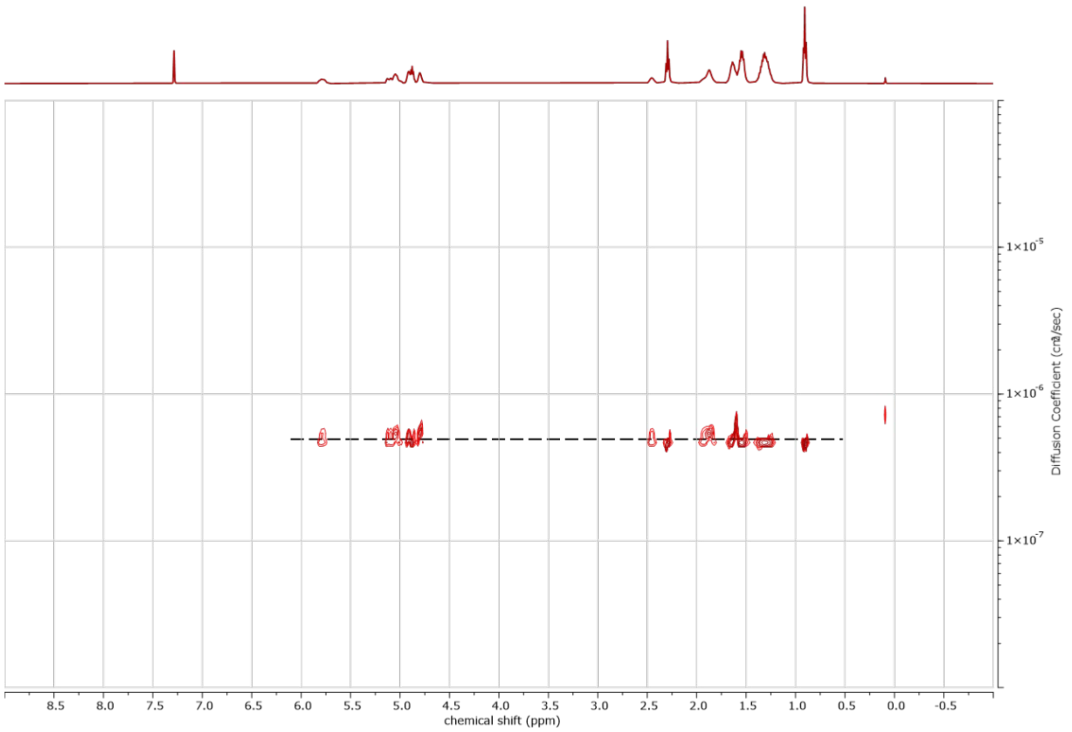

Figure S16. ^{1}H DOSY NMR spectrum (500 MHz, $CDCl_3$) of $(AB)_3$-88.

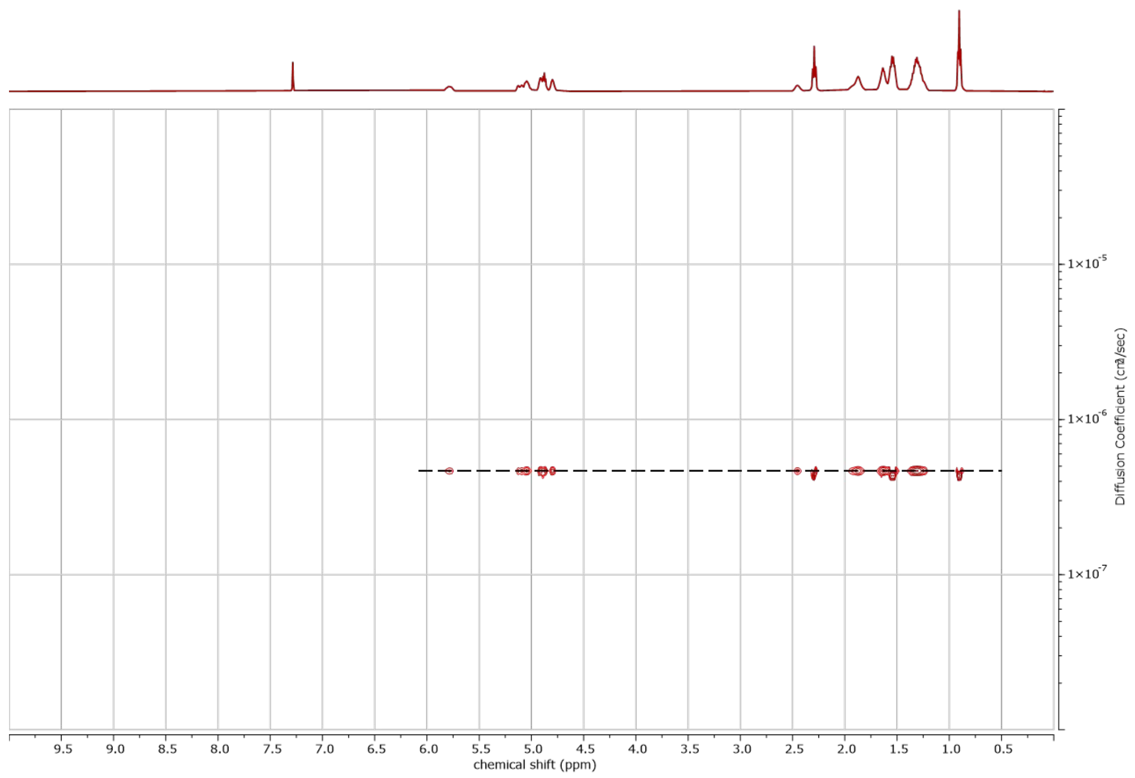

Figure S17. ^{1}H DOSY NMR spectrum (500 MHz, $CDCl_3$) of $(AB)_4$-90.

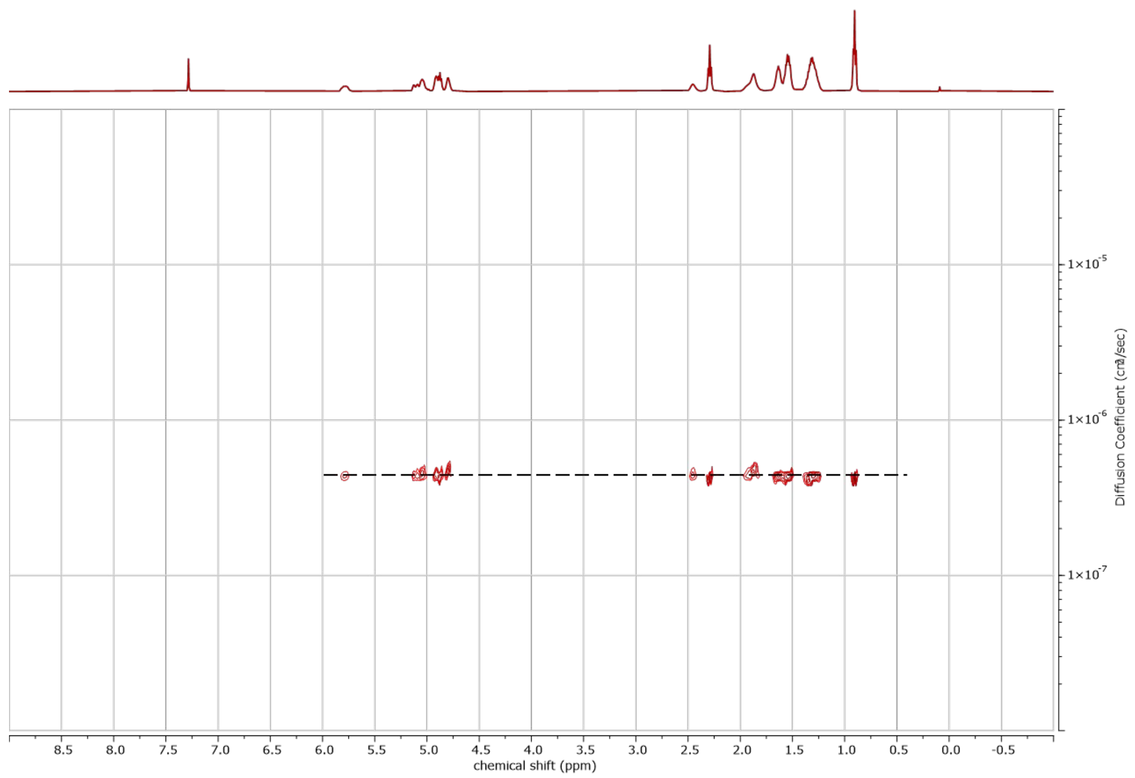

Figure S18. ^{1}H DOSY NMR spectrum (500 MHz, $CDCl_3$) of $(AB)_6$-72.

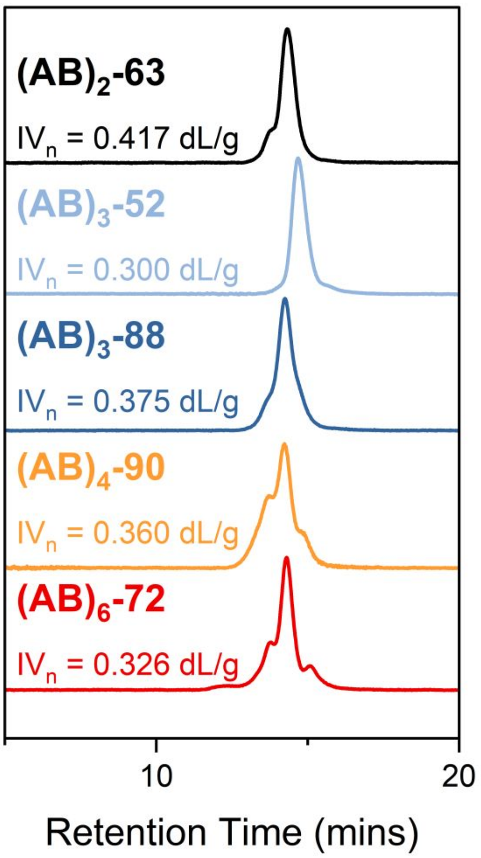

Figure S19. Viscometry SEC elution trace of $(AB)_n$ star block polymers and number average intrinsic viscosities.

While monomer feed ratios were held constant, the PER and DPE CTAs used to initiate the polymerizations were recrystallized from water and thus may contain varying, but critically small, amounts of residual protic impurities. These protic species can lead to occasional chain-transfer events during ROP or ROCOP, resulting in trace quantities of short-chain by-products. Because each polymerization used a different multi-functional CTA, slight variations in impurity content are expected, which explains the minor differences in shoulder intensity. Importantly, these features represent a small fraction of the overall distribution, and the dominant elution peak in each case corresponds to the expected star block copolymer. Future work should focus on the use of CTAs not recrystalized in water to avoid this issue.

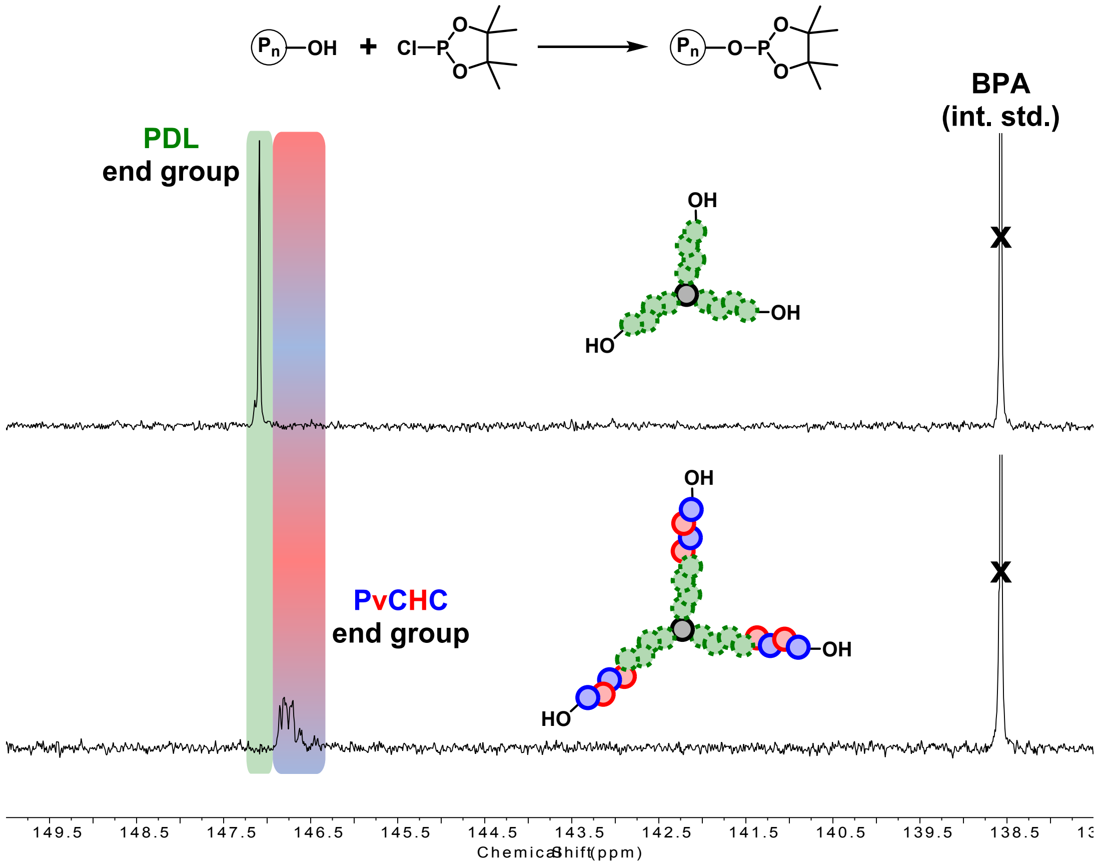

Figure S20. $^{31}P\{^{1}H\}$ NMR spectra comparing $(PDL)_3$ and $(AB)_3$ (A = PvCHC, B = PDL) end-group titrations.

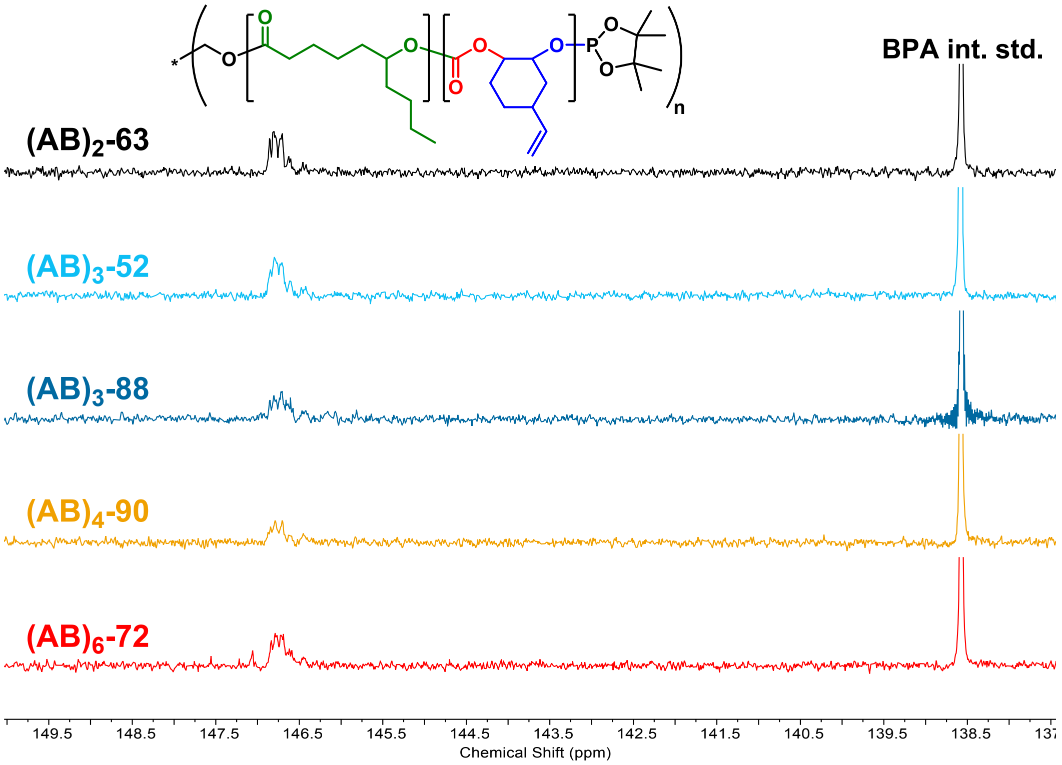

Figure S21. $^{31}P\{^{1}H\}$ NMR end-group analysis of $(AB)_n$-# star block polymers.

Table S3. Thermogravimetric Analysis of $(AB)_n$ Unmodified and $(A^{OH}B)_n$ Modified Star Block Polymers

Entry	Polymer[a]	$T_{d,5\%}$[b] (°C)
1	$(AB)_2$-63	256
2	$(AB)_3$-52	269
3	$(AB)_3$-88	258
4	$(AB)_4$-90	266
5	$(A^{OH}B)_2$-63	245
6	$(A^{OH}B)_3$-52	235
7	$(A^{OH}B)_3$-88	222
8	$(A^{OH}B)_4$-90	222

[a]$(A^{OH}B)_n$-#; A^{OH} = OH-functional PvCHC, B = PDL, n = number of arms, # = molar mass (SEC). [b] Determined by TGA. Reported as temperature at 5% mass loss.

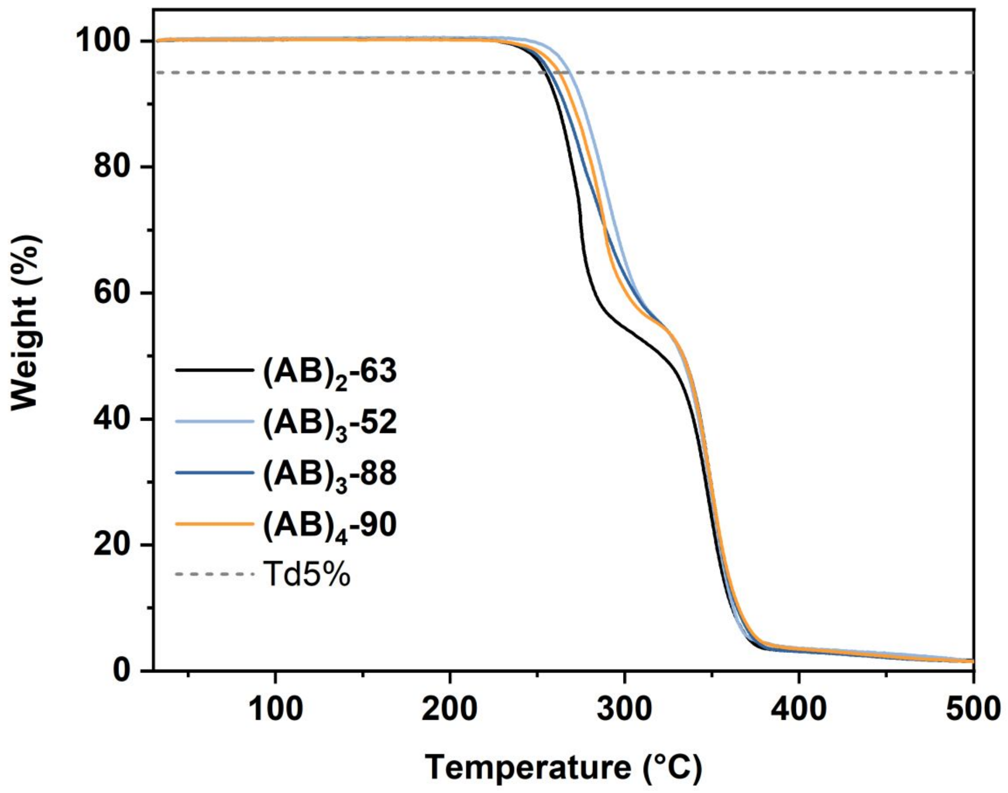

Figure S22. TGA profile for $(AB)_n$ unmodified star block polymers.

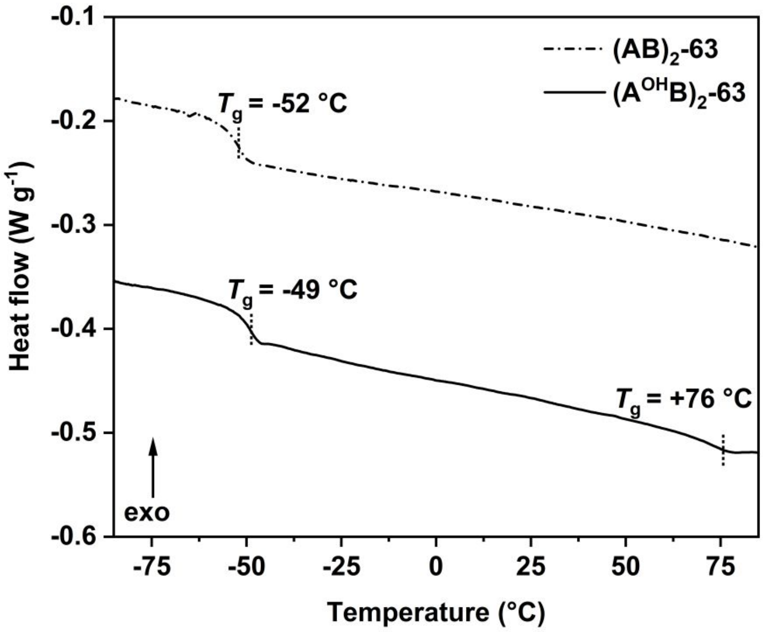

Figure S23. DSC thermograms for 2-arm star block polymer samples $(AB)_2$-63 (non-modified) and $(A^{OH}B)_2$-63 (modified).

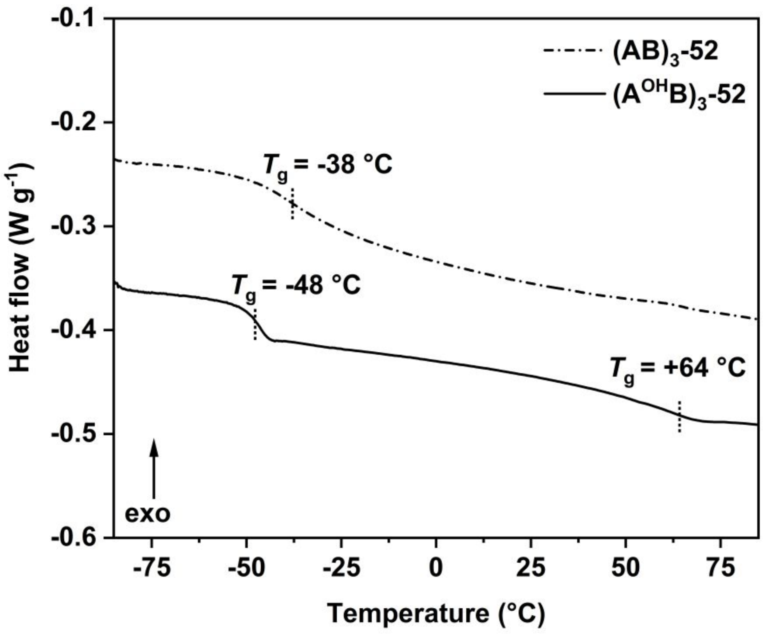

Figure S24. DSC thermograms for 3-arm star block polymer samples $(AB)_3$-52 (non-modified) and $(A^{OH}B)_3$-52 (modified).

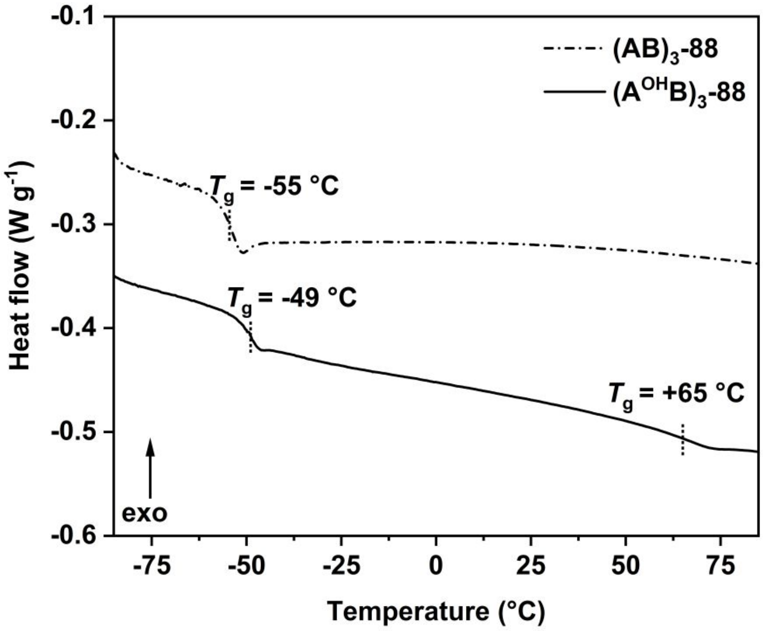

Figure S25. DSC thermograms for 3-arm star block polymer samples (AB)3-88 (non-modified) and (A^{OH}B)3-88 (modified).

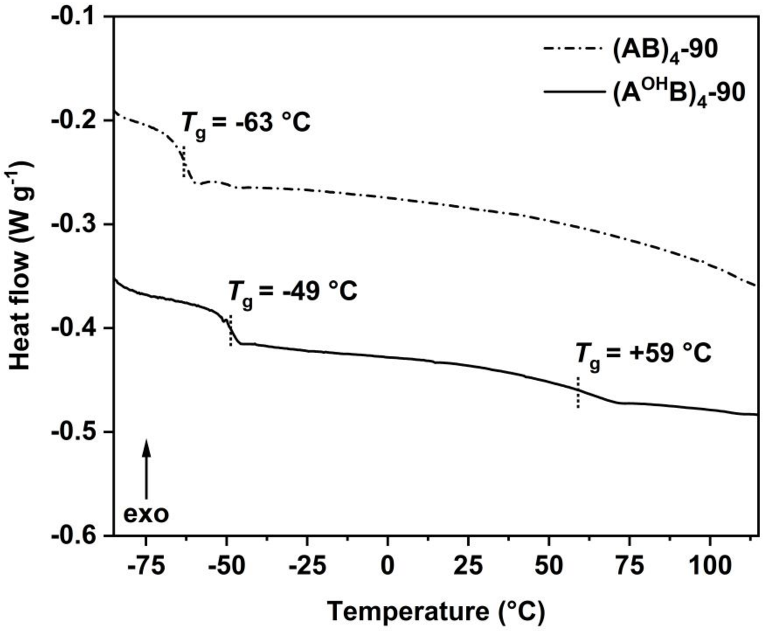

Figure S26. DSC thermograms for 4-arm star block polymer samples (AB)$_4$-90 (non-modified) and (A^{OH}B)$_4$-90 (modified).

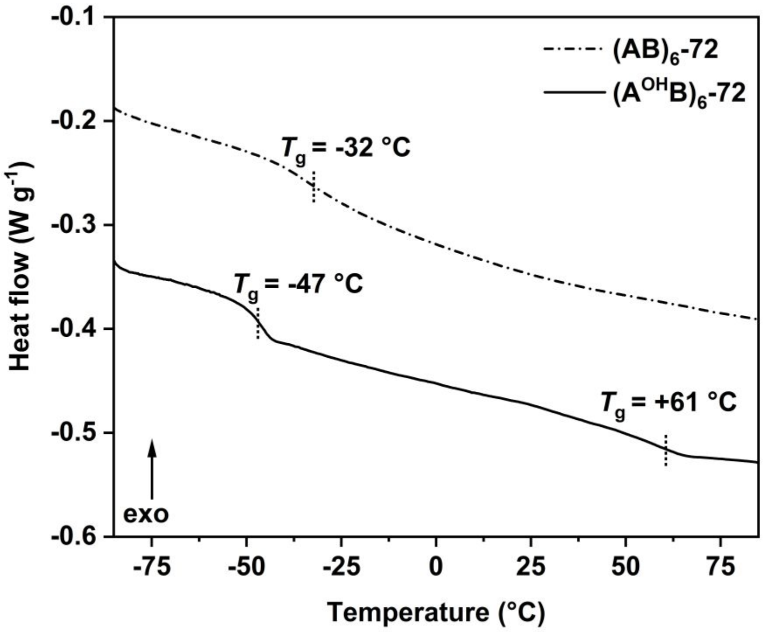

Figure S27. DSC thermograms for 6-arm star block polymer samples $(AB)_6$-72 (non-modified) and $(A^{OH}B)_6$-72 (modified).

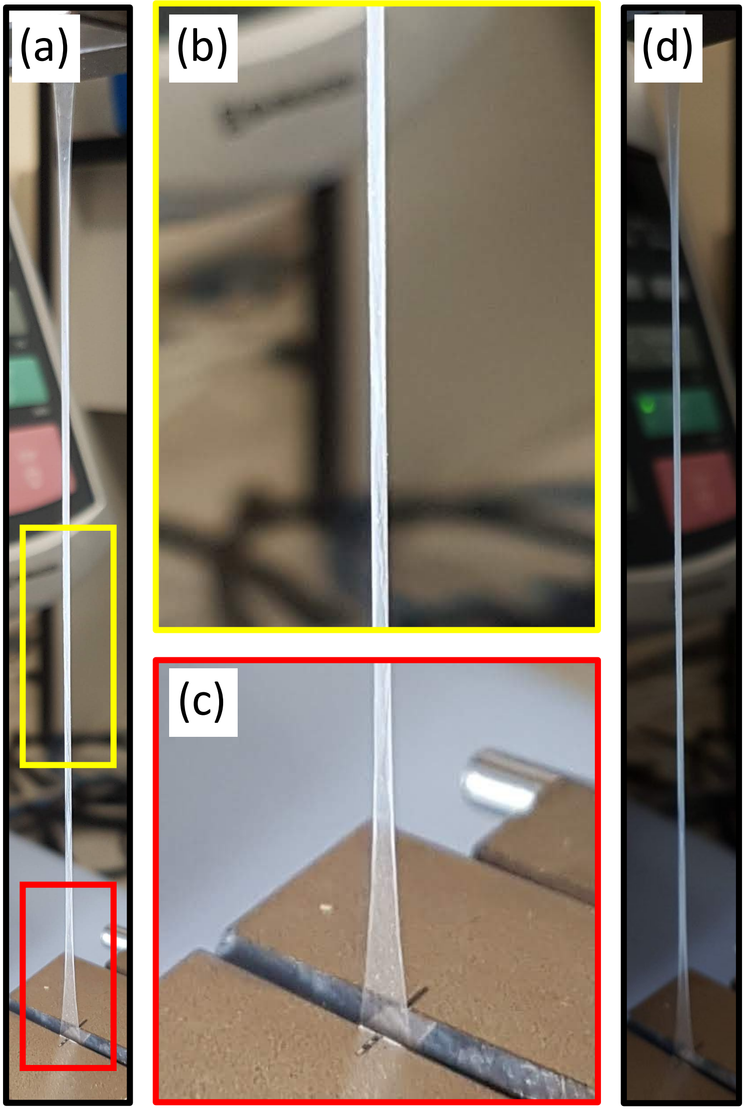

Figure S28. Digital photographs of sample $(AB)_2$-63 during uniaxial extension: (a) Full view of specimen with areas of interest marked; (b) Enlarged view of yellow highlighted area showing whitening of the central specimen area; (c) Enlarged view of red highlighted area showing lower extent of whitening nearer the specimen shoulder (clamped) area; (d) Full view of specimen with background light darkened to highlight contrast of stress-whitening.

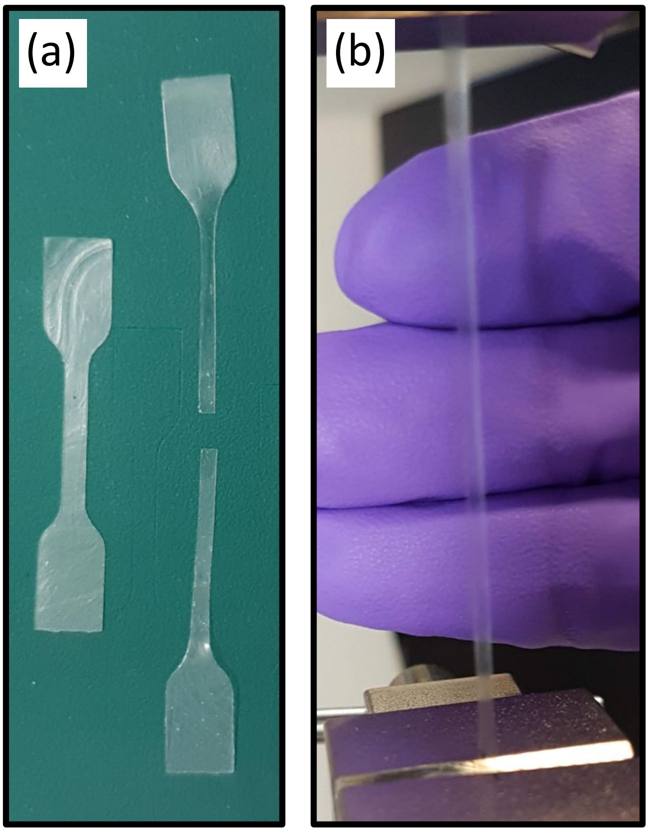

Figure S29. Digital photographs of sample $(AB)_3$-88: (a) Pristine and fractured test specimens before and after uniaxial extension, respectively; (b) Central region of a sample during uniaxial extension with absence of stress-whitening.

Table S4. $(A^{OH}B)_n$ Modified Star Block Polymer Thermal Characterisation Data

Entry	Polymer[a]	$T_{g,DSC}$[b] (°C)	$T_{g,DMTA}$[c] (°C)
1	$(A^{OH}B)_2$-63	-49; +76	+99
2	$(A^{OH}B)_3$-52	-48; +64	+90
3	$(A^{OH}B)_3$-88	-49; +65	+95
4	$(A^{OH}B)_4$-90	-49; +59	+87
5	$(A^{OH}B)_6$-72	-47; +61	+79

[a]$(A^{OH}B)_n$-#; A^{OH} = OH-functional PvCHC, B = PDL, n = number of arms, # = molar mass (SEC). [b]Glass transition temperatures determined by DSC (-90 – 120 °C) from the midpoint of transition in the third heating cycle. [c]Upper glass transition temperature determined from a DMTA thermal sweep (30 – 110 °C), taken as the peak maxima in tan(δ).

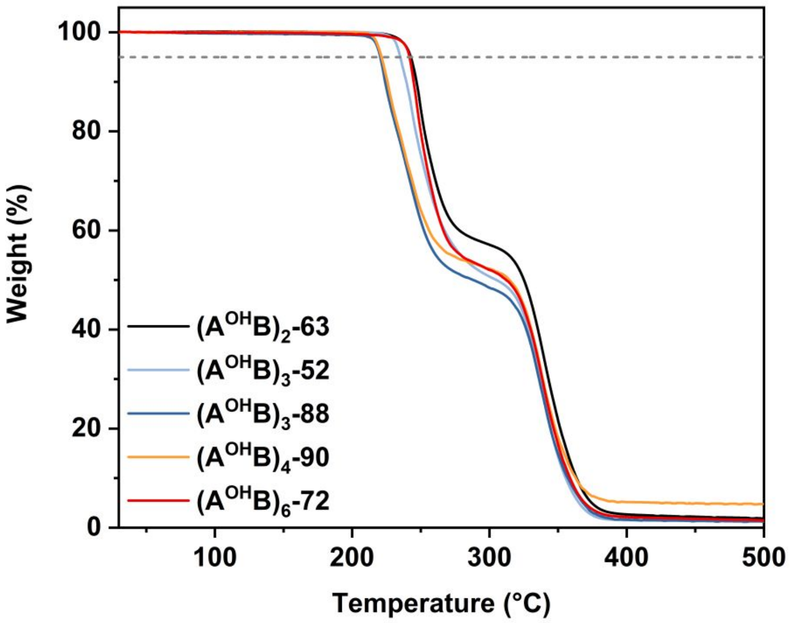

Figure S30. TGA profile for $(A^{OH}B)_n$ modified star block polymers.

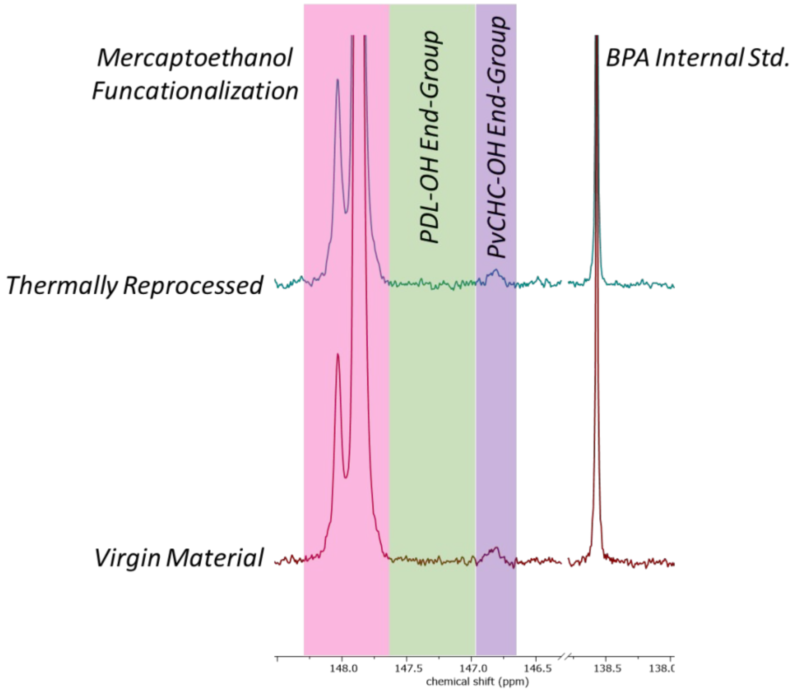

Figure S31. $^{31}P\{^{1}H\}$ NMR end-group analysis of virgin and thermally reprocessed (120 ˚C, 1 ton m^{-2}, 30 mins) $(A^{OH}B)_3$-88.

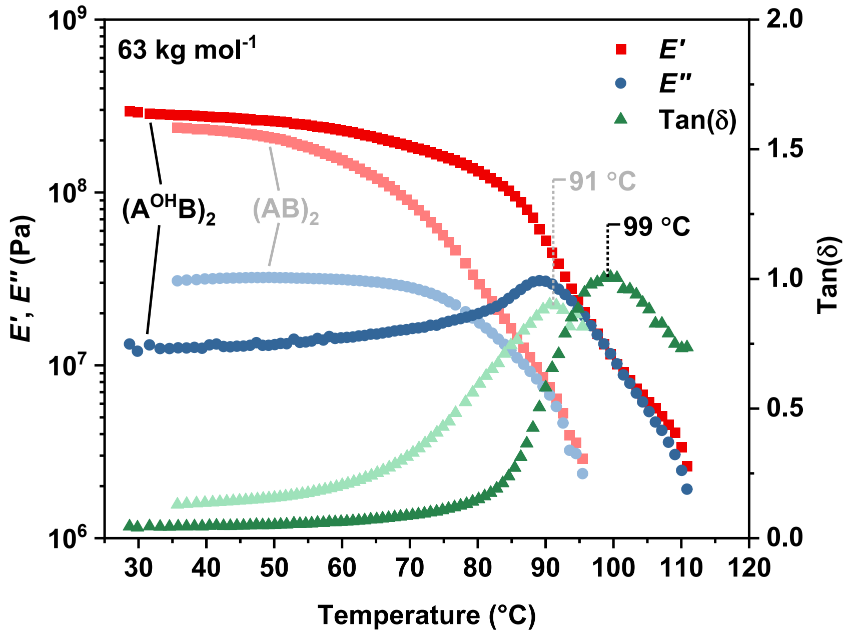

Figure S32. DMA temperature sweep profiles for star block polymer samples $(AB)_2$-63 (non-modified) and $(A^{OH}B)_2$-63 (modified).

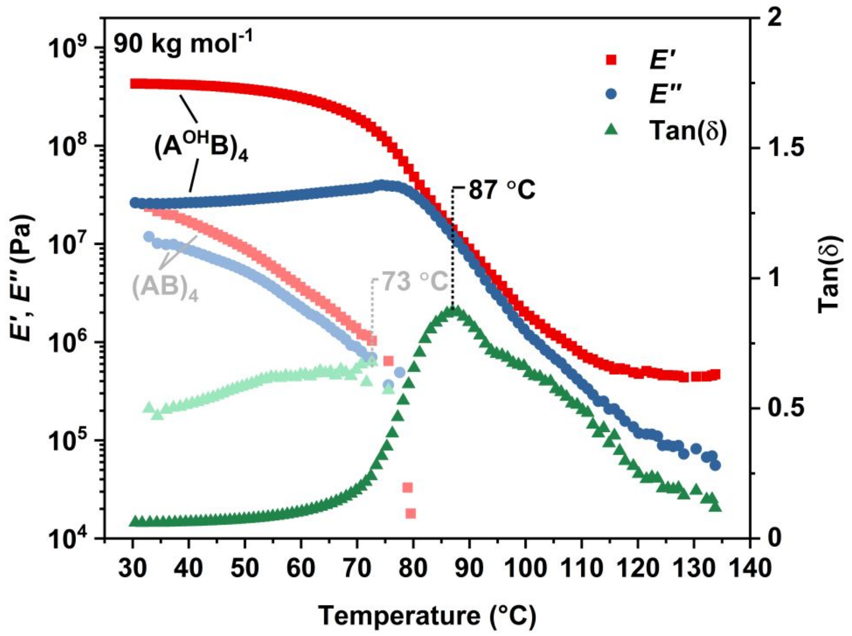

Figure S33. DMA temperature sweep profiles for star block polymer samples $(AB)_4$-90 (non-modified) and $(A^{OH}B)_4$-90 (modified).

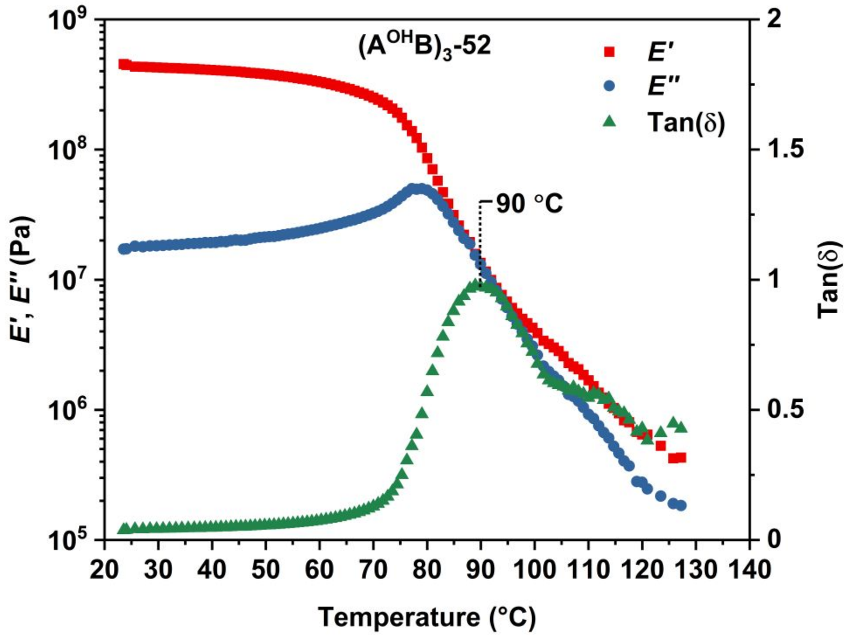

Figure S34. DMA temperature sweep profiles for star block polymer sample $(A^{OH}B)_3$-52 (modified).

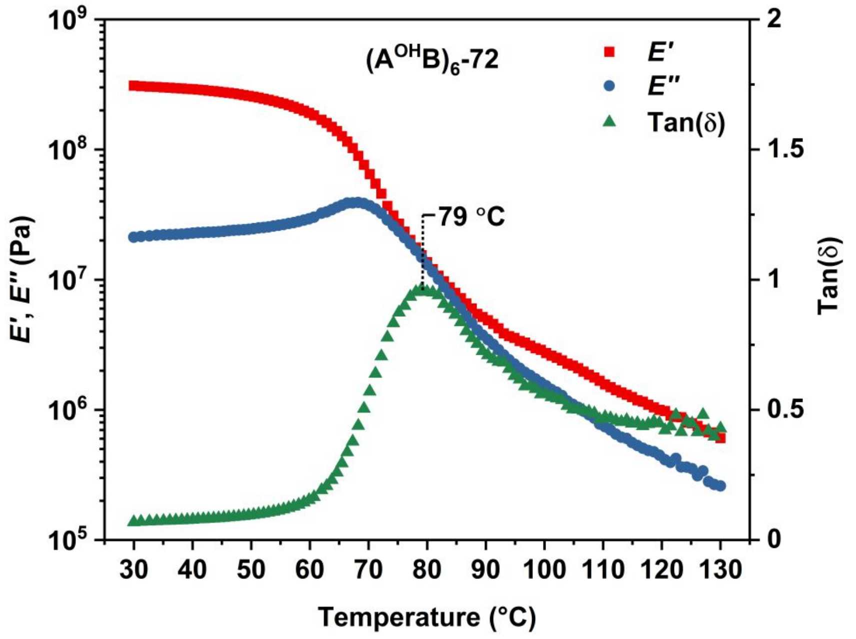

Figure S35. DMA temperature sweep profiles for star block polymer sample $(A^{OH}B)_6$-72 (modified).

Table S5. $(A^{OH}B)_n$ Modified Star Block Polymer Mechanical Characterisation Data

Entry	Polymer	E_y[a] (MPa)	σ_y[b] (MPa)	ε_y[c] (%)	σ[d] (MPa)	ε_b[e] (%)
1	$(A^{OH}B)_2$-63	172 ± 23	5.3 ± 1.1	5.4 ± 0.8	10 ± 1.5	550 ± 205
2	$(A^{OH}B)_3$-52	165 ± 15	7.4 ± 0.6	7.2 ± 1.2	12 ± 1.0	1051 ± 115
3	$(A^{OH}B)_3$-88	186 ± 20	6.2 ± 1.5	5.9 ± 1.1	12 ± 4.0	661 ± 254
4	$(A^{OH}B)_4$-90	169 ± 10	6.9 ± 0.3	6.3 ± 0.3	8.5 ± 0.6	737 ± 67
5	$(A^{OH}B)_6$-72	156 ± 13	6.5 ± 0.5	8.3 ± 1.0	11 ± 1.4	615 ± 161

[a]Young's modulus. [b]Yield stress. [c]Yield strain. [d]Tensile strength. [e]Elongation at break. Average values and standard deviation from a minimum of 5 specimens.

Table S6. Small-Angle X-ray Scattering (SAXS) Data of $(A^{[OH]}B)_n$ Star Block Polymers

Entry	Polymer	Observed q/q^*[a]	q^*[b] (nm^{-1})	d^*[c] (nm)	Morphology[d]
		**Non-modified**			
1	$(AB)_2$-63	1	0.332	18.9	*n.d.*
2	$(AB)_3$-52	1	0.452	13.9	*n.d.*
3	$(AB)_3$-88	1	0.375	16.8	*n.d.*
4	$(AB)_4$-90	1	0.438	14.3	*n.d.*
		**Modified**			
5	$(A^{OH}B)_2$-63	1, 2, 3, (4)	0.206	30.5	LAM
6	$(A^{OH}B)_3$-52	1, 2, 3, 4, 5	0.314	20.0	LAM
7	$(A^{OH}B)_3$-88	1, 2, 3, (4)	0.238	26.4	LAM
8	$(A^{OH}B)_4$-90	1, (2), 3	0.293	21.4	LAM

[a]Observed Bragg reflections, brackets indicate broad and/or near-absent higher-order reflections. [b]Principal scattering peak. [c]Domain spacing, calculated from the principal scattering peak, $d^* = 2\pi/q^*$. [d]Phase morphology (domain microstructure) assigned according to SAXS data; LAM = lamellar, *n.d.* = no assignment possible due to absence of higher order scattering peaks, likely weakly ordered/disordered. See Appendix Figures S7.62 – 7.69 for 1D SAXS patterns.

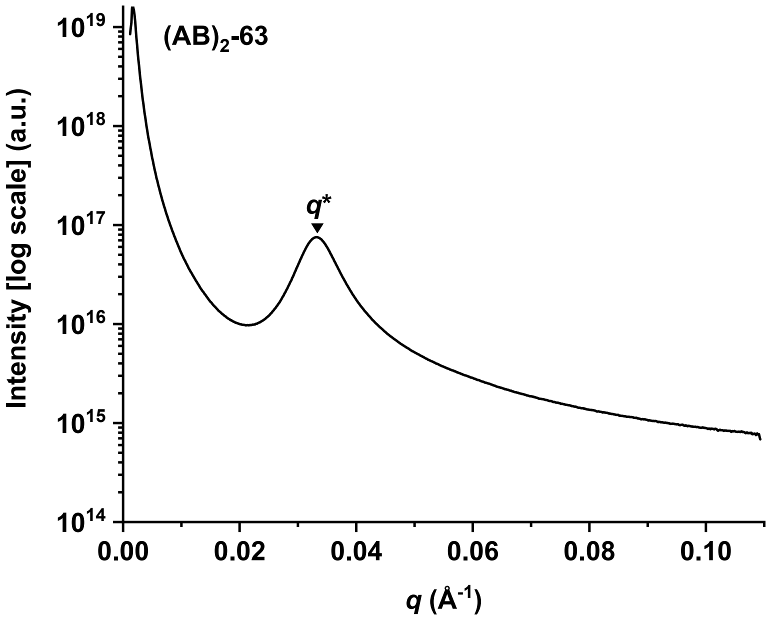

Figure S36. 1D SAXS profile for 2-arm star block polymer sample $(AB)_2$-63.

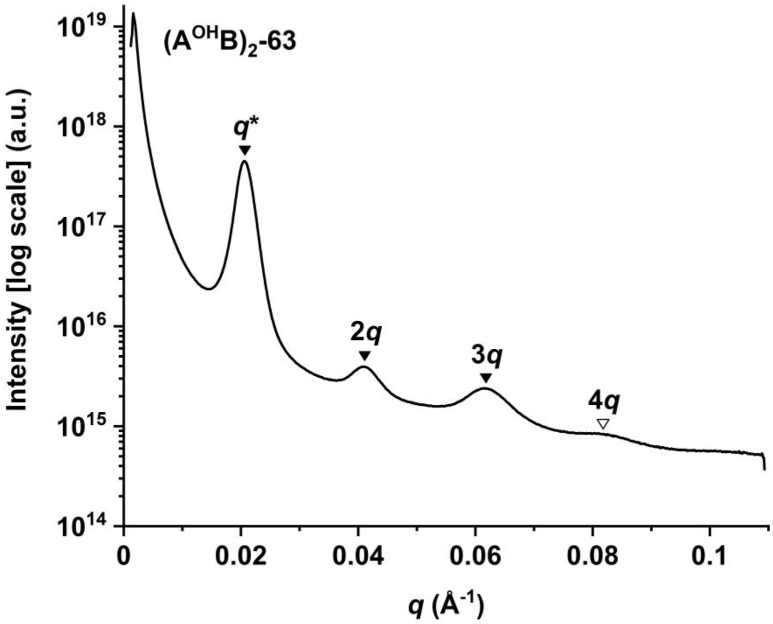

Figure S37. 1D SAXS profile for hydroxyl-modified 2-arm star block polymer sample $(A^{OH}B)_2$-63.

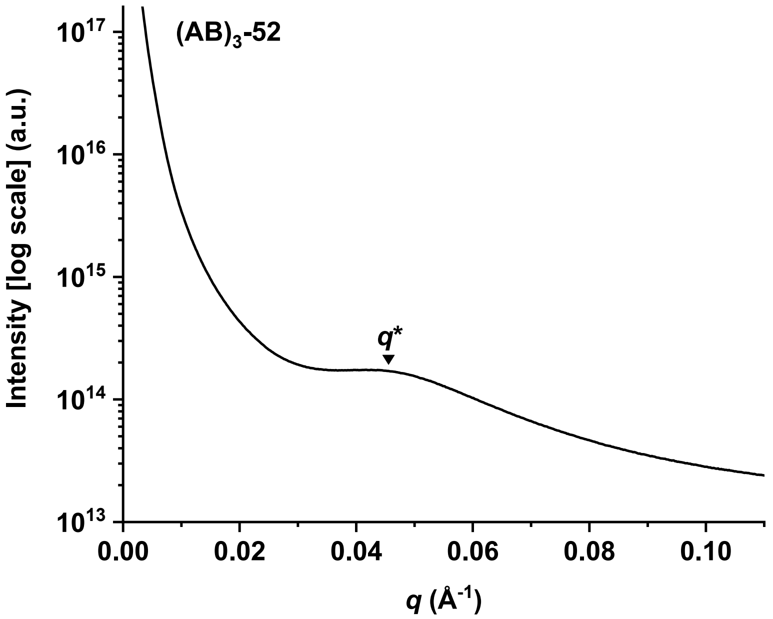

Figure S38. 1D SAXS profile for 3-arm star block polymer sample (AB)$_3$-52.

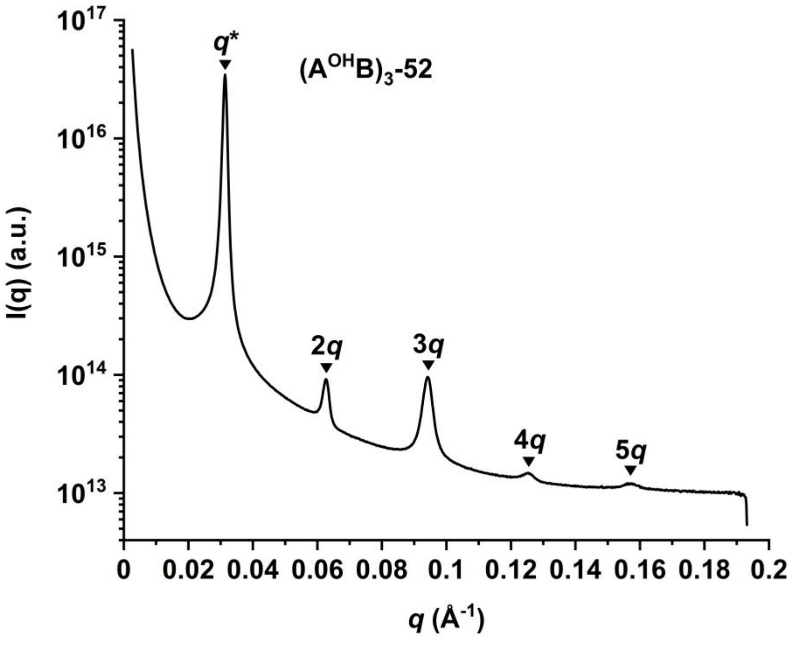

Figure S39. 1D SAXS profile for hydroxyl-modified 3-arm star block polymer sample (A^{OH}B)$_3$-52.

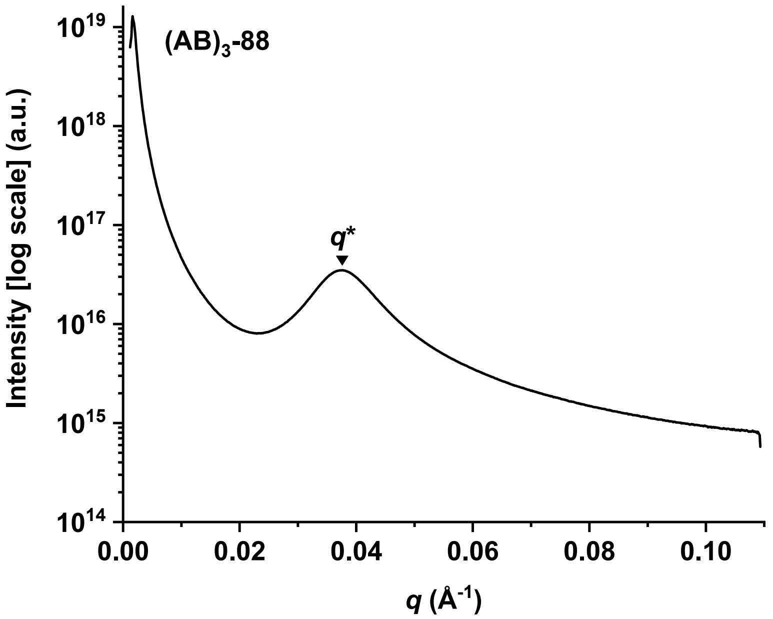

Figure S40. 1D SAXS profile for 3-arm star block polymer sample $(AB)_3$-88.

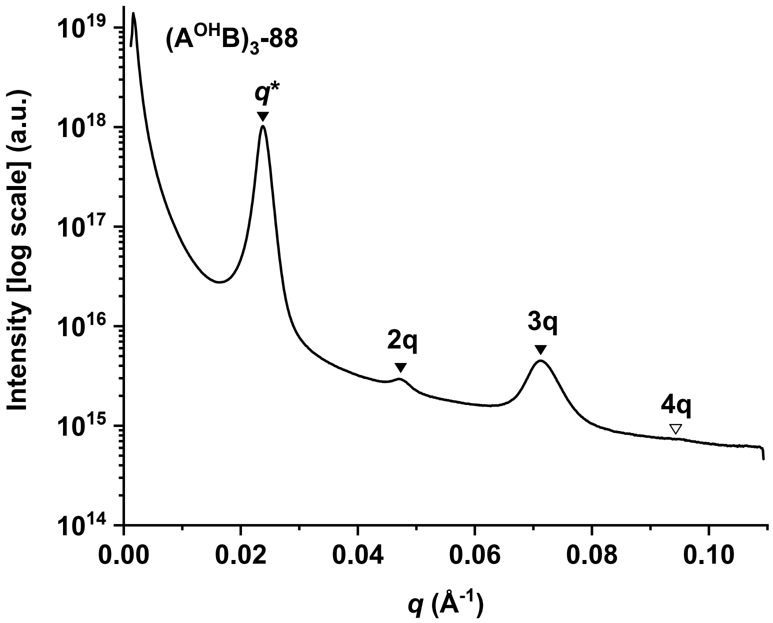

Figure S41. 1D SAXS profile for hydroxyl-modified 3-arm star block polymer sample $(A^{OH}B)_3$-88.

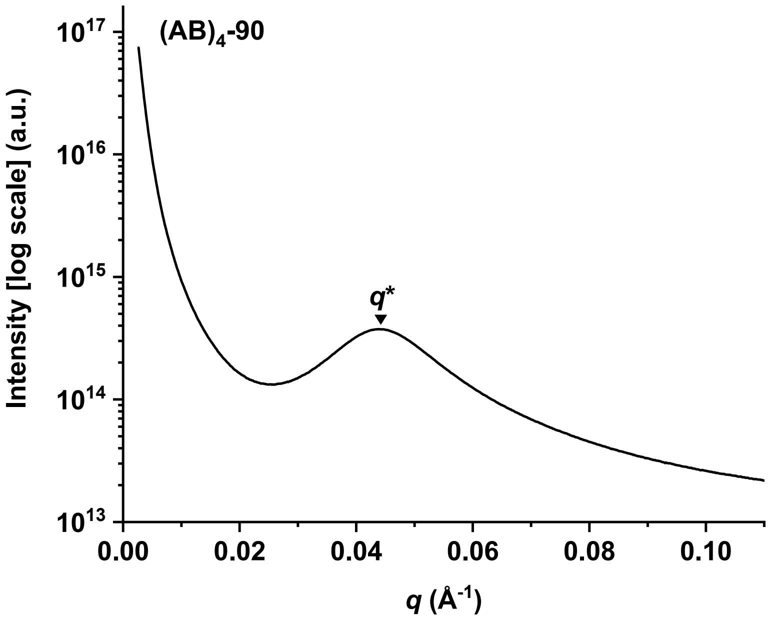

Figure S42. 1D SAXS profile for 4-arm star block polymer sample $(AB)_4$-90.

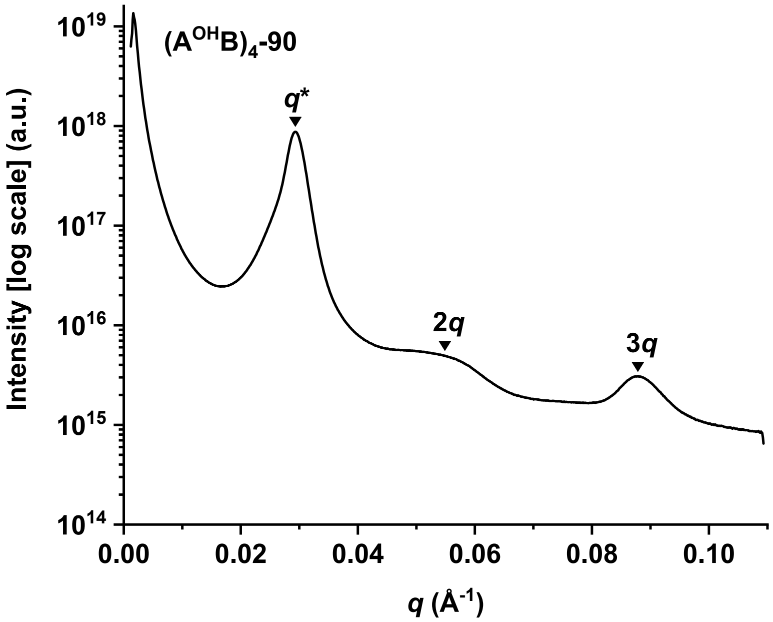

Figure S43. 1D SAXS profile for hydroxyl-modified 4-arm star block polymer sample $(A^{OH}B)_4$-90.

References

(1) Kember, M. R.; Knight, P. D.; Reung, P. T. R.; Williams, C. K. Highly Active Dizinc Catalyst for the Copolymerization of Carbon Dioxide and Cyclohexene Oxide at One Atmosphere Pressure. *Angew. Chem. Int. Ed.* **2009**, *48* (5), 931-933. DOI: 10.1002/anie.200803896.

(2) Filik, J.; Ashton, A. W.; Chang, P. C. Y.; Chater, P. A.; Day, S. J.; Drakopoulos, M.; Gerring, M. W.; Hart, M. L.; Magdysyuk, O. V.; Michalik, S.; et al. Processing two-dimensional X-ray diffraction and small-angle scattering data in DAWN 2. *J. Appl. Crystallogr.* **2017**, *50* (Pt 3), 959-966. DOI: 10.1107/s1600576717004708 From NLM.

(3) Pauw, B. R.; Smith, A. J.; Snow, T.; Terrill, N. J.; Thünemann, A. F. The modular small-angle X-ray scattering data correction sequence. *J. Appl. Crystallogr.* **2017**, *50* (Pt 6), 1800-1811. DOI: 10.1107/s1600576717015096 From NLM.

(4) Sulley, G. S.; Gregory, G. L.; Chen, T. T. D.; Peña Carrodeguas, L.; Trott, G.; Santmarti, A.; Lee, K.-Y.; Terrill, N. J.; Williams, C. K. Switchable Catalysis Improves the Properties of CO2-Derived Polymers: Poly(cyclohexene carbonate-b-ε-decalactone-b-cyclohexene carbonate) Adhesives, Elastomers, and Toughened Plastics. *J. Am. Chem. Soc.* **2020**, *142* (9), 4367-4378. DOI: 10.1021/jacs.9b13106.

(5) Romain, C.; Garden, J. A.; Trott, G.; Buchard, A.; White, A. J. P.; Williams, C. K. Di-Zinc–Aryl Complexes: CO2 Insertions and Applications in Polymerisation Catalysis. *Chem. Eur. J.* **2017**, *23* (30), 7367-7376. DOI: 10.1002/chem.201701013.

(6) Spyros, A.; Argyropoulos, D. S.; Marchessault, R. H. A Study of Poly(hydroxyalkanoate)s by Quantitative 31P NMR Spectroscopy: Molecular Weight and Chain Cleavage. *Macromolecules* **1997**, *30*, 327-329.

(7) Spyros, A.; Argyropoulos, D. S.; Marchessault, R. H. A Study of Poly(hydroxyalkanoate)s by Quantitative 31P NMR Spectroscopy: Molecular Weight and Chain Cleavage. *Macromolecules* **1997**, *30* (2), 327-329. DOI: 10.1021/ma9601979.
